# Trim33 Binds and Silences a Class of Young Endogenous Retroviruses in the Mouse Testis; a Novel Component of the Arms Race between Retrotransposons and the Host Genome

**DOI:** 10.1371/journal.pgen.1005693

**Published:** 2015-12-01

**Authors:** Luke Isbel, Rahul Srivastava, Harald Oey, Alex Spurling, Lucia Daxinger, Hamsa Puthalakath, Emma Whitelaw

**Affiliations:** Department of Biochemistry and Genetics, La Trobe Institute for Molecular Science, La Trobe University, Bundoora, Melbourne, Australia; University of British Columbia, CANADA

## Abstract

Transposable elements (TEs) have been active in the mammalian genome for millions of years and the silencing of these elements in the germline is important for the survival of the host. Mice carrying reporter transgenes can be used to model transcriptional silencing. A mutagenesis screen for modifiers of epigenetic gene silencing produced a line with a mutation in *Trim33;* the mutants displayed increased expression of the reporter transgene. ChIP-seq of Trim33 in testis revealed 9,109 peaks, mostly at promoters. This is the first report of ChIP-seq for Trim33 in any tissue. Comparison with ENCODE datasets showed that regions of high read density for Trim33 had high read density for histone marks associated with transcriptional activity and mapping to TE consensus sequences revealed Trim33 enrichment at RLTR10B, the LTR of one of the youngest retrotransposons in the mouse genome, MMERVK10C. We identified consensus sequences from the 266 regions at which Trim33 ChIP-seq peaks overlapped RLTR10B elements and found a match to the A-Myb DNA-binding site. We found that TRIM33 has E3 ubiquitin ligase activity for A-MYB and regulates its abundance. RNA-seq revealed that mice haploinsufficient for Trim33 had altered expression of a small group of genes in the testis and the gene with the most significant increase was found to be transcribed from an upstream RLTR10B. These studies provide the first evidence that A-Myb has a role in the actions of Trim33 and suggest a role for both A-Myb and Trim33 in the arms race between the transposon and the host. This the first report of any factor specifically regulating RLTR10B and adds to the current literature on the silencing of MMERVK10C retrotransposons. This is also the first report that A-Myb has a role in the transcription of any retrotransposon.

## Author Summary

Almost half of the genomes of humans and mice are made up of transposable elements. During host evolution, subsets of these elements have periods of transpositional activity during which they spread throughout the genome. This is dependent on the transcriptional activity of these elements in the cells that contribute to the germline. Hosts have evolved pathways to silence their expression. A number of Trim family proteins have been found to have a role in silencing transposable elements, and it was previously shown that Trim33 shared this function in liver. However, the function of Trim33 in other tissues is poorly understood. Here we report a role for Trim33 in silencing a specific subset of retrotransposons that contain RLTR10B LTRs, in the germline. We also show the transcription factor, A-Myb, is responsible for activating transcription of these elements and it is likely that a subset of RLTR10Bs have recently evolved Myb DNA binding sites to capitalise on the critical role that the A-Myb transcription factor has in germ cells. Suppression of A-Myb activity by Trim33 provides a plausible mechanism by which the host keeps transposons in check.

## Introduction

Approximately half of the genomes of humans and mice are made up of transposable elements (TEs) and numerous pathways, both genetic and epigenetic, have evolved to repress their expression. Each TE has had a period of transpositional activity during which it spreads through the genome and this is dependent on its transcriptional activity in the germline. It has been suggested that during periods of epigenetic reprogramming, such as occurs in the germline, when DNA methylation is low, other mechanisms are used to suppress the expression of recently transposed retrotransposons [1]. Because of the difficulty of mapping individual repeats back to the genome, the epigenetic and transcriptional state of retrotransposons has been difficult to study. Improved next generation sequencing chemistry, resulting in longer reads, is helping to overcome this problem and bioinformatics tools are being refined to deal with repetitive elements.

Unbiased genetic screens for modifiers of epigenetic gene silencing have been carried out in a number of model organisms, including the mouse, and have provided a valuable tool in the identification of the proteins responsible for mediating transcriptional silencing of inserted reporter transgenes [2, 3]. The silencing of transgenes is thought to mimic, in many ways, that of retrotransposons [4, 5]. We have carried out a dominant mouse screen using a line containing a GFP transgene array that undergoes stochastic silencing in erythroid cells and the alleles identified are termed *Modifiers of murine metastable epialleles Dominant* (*MommeDs*) [4]. The underlying genes responsible for many but not all of the *MommeD* lines have been identified and reported; *MommeD1*,*2*,*4*,*5*,*8–10*,*12–14*,*16–19*,*23*,*27*,*28*,*30–40*,*4*2 [3], *MommeD6*,*8* [6] and the results of the screen have recently been reviewed [7]. The genes encode known epigenetic modifiers, including DNA methyltransferases, chromatin remodellers, histone methyltransferases, histone deacetylases and some previously uncharacterised proteins, such as Smchd1 and Rlf. Here we report, for the first time, the mutation underlying *MommeD44* and the consequences of this mutation on the transcriptome in the mouse.

*MommeD44* heterozygous mutants showed an increase in the expression of the reporter transgene compared to that of their wildtype littermates. The underlying *N-ethyl-N-nitrosourea*-induced mutation was found to have produced a null allele of the gene *Trim33*, *Tripartite motif containing 33*, also known as *Tif1γ*, which codes for a protein that contains a tripartite motif with ubiquitin ligase activity and two chromatin binding domains; a bromodomain and a PHD (plant homodomain) finger. The function of Trim33 remains poorly understood. In cell culture, Trim33 can act as transcriptional coregulator via the TGFβ pathway [8] and mice homozygous for a null allele die during embryonic development [9]. While few functional studies have been carried out in whole animals, it has been shown that Trim33 can act in combination with the close homologues, Trim24 and Trim28; Lox-Cre knockdown of *Trim33* in the liver of mice null for *Trim24* results in increased expression of the VL30 class of retrotransposons [10] and hepatocellular carcinoma [11]. Unlike Trim28, which is known to recruit chromatin modifying protein complexes to the 5’ UTR of ERVs [12], the mechanism by which Trim33 acts has been unclear.

Trim33 has been shown to be highly expressed in the spermatogonia and primary spermatocytes of the testis [13] but its role in these cells is unknown. We have carried out ChIP-seq in adult testis and show widespread binding to active gene promoters, as well as to the members of the RLTR10B class of retrotransposon that are marked by H3K27ac. Using publically available datasets, we find considerable overlap between Trim33 and A-Myb binding sites. Transcriptome analysis, using heterozygous *MommeD44* mice, demonstrates a role for Trim33 as a dominant transcriptional repressor of RLTR10B elements in the testis and a combination of bioinformatics and biochemical studies suggests that this repression involves the A-Myb transcription factor. This is the first report of ChIP-seq for Trim33 and the first report of a function for Trim33 in the testis.

## Results

### Identification of the *MommeD44* mutant and the underlying mutation

**Fig 1:**
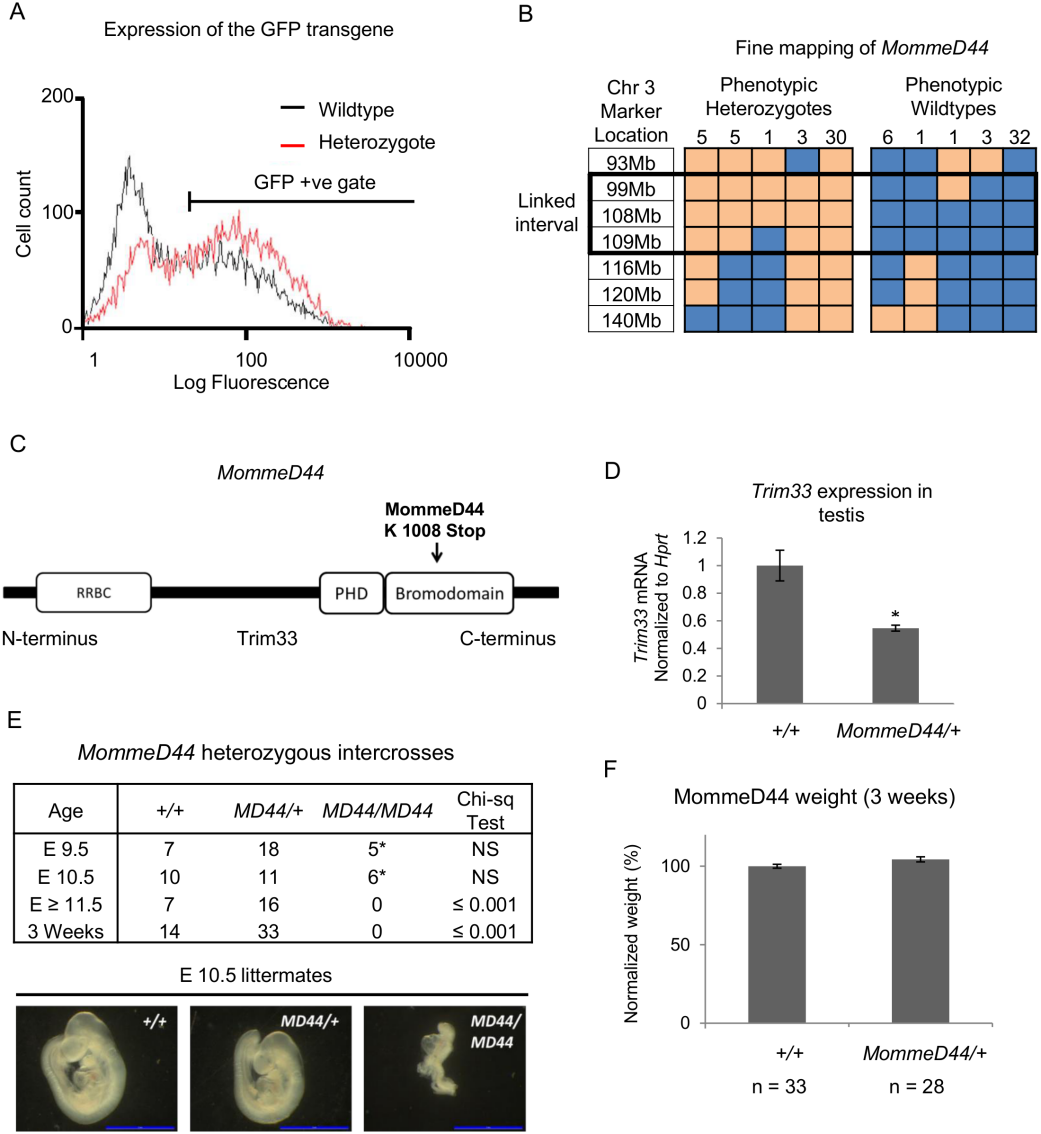
The *Momme44* mutation produces a null allele of *Trim33*. (**A**) Representative GFP profiles for *MommeD44* wildtype (black) and heterozygous (red) mice are shown by the numbers of erythrocytes expressing GFP along a log scale of fluorescence. A GFP gate shows cells positive for GFP expression. (**B**) Genetic mapping of phenotypically heterozygous and wildtype F2 (C57/FVB) mice indicates the boundaries for the *MommeD44* mutation. FVB/C57 genotype SNPs are shaded yellow and C57/C57 genotype SNPs are shaded blue. Numbers of mice representing each SNP profile are indicated. (**C**) The *MommeD44* mutation is located in the Bromodomain of Trim33 and changes a leucine to a stop codon. (**D**) Mice heterozygous for the *MommeD44* mutation had significantly reduced levels of *Trim33* mRNA, normalized to the housekeeping gene *Hprt*, in the testis. * p value ≤ 0.05, error bars indicate SEM, n ≥ 3 mice per genotype. (**E**) Embryos from heterozygous intercrosses show that *MommeD44* homozygous embryos die during early development. Astericies indicate abnormal embryos, represented by E10.5 *MD44/MD44* image. (**F**) The weight of *MommeD44* heterozygous mice (*MD44*/*+*) at weaning. The weight of each mouse was normalized to the average weight of wildtype mice from each litter, error bars indicate SEM,* p value ≤ 0.05.

The experimental pipeline for the screen has been described previously [3]. The mutagenesis was carried out in the FVB/NJ background and the *MommeD44* founder was identified because it showed an increase in the proportion of red blood cells expressing the GFP reporter (Fig 1A). As such, *MommeD44* was classified as a suppressor of variegation. This readout was used to maintain the *MommeD44* allele for five generations prior to performing genetic linkage and exome sequencing. Mapping was carried out following a F2 backcross to *Line3C* (a C57BL/6J strain carrying the same GFP transgene array at the same location), using the general method described previously[6]. Using the Illumina GoldenGate SNP genotyping assay, we identified an interval on Chr3 between 75Mb and 155Mb (S1 Fig). Fine mapping, using additional mice, reduced the interval to Chr3 between 99Mb and 109Mb (Fig 1B).

To identify the underlying mutation, whole exome deep sequencing of DNA was carried out and variants were called within the linked interval. An exonic mutation was identified in the *Trim33* gene, which is located in the linked interval. The A → T mutation changes a lysine to a premature stop codon (Fig 1C). We designate this allele *Trim33* ^*MommeD44*^ and genotyping mice from the colony showed that the presence of the mutation correlated well with the altered GFP profile (S2 Fig). RTqPCR was carried out to determine the effect of the mutation on the level of *Trim33* mRNA (Fig 1D). The study was carried out in testis because *Trim33* is expressed in that tissue at much higher levels than in any other tissue in the adult [13]. Heterozygotes have approximately half the *Trim33* mRNA level seen in wildtypes, suggesting that the mutant transcript undergoes nonsense mediated mRNA decay. Using an antibody that binds specifically to Trim33 (S3A Fig), a similar decrease in the level of Trim33 protein was found in heterozygous mutant testis (S3B and S3C Fig) and no band can be seen in homozygous mutant embryos (S3D Fig).

We examined the viability of homozygous mutants. As expected, embryos homozygous for the mutation were grossly abnormal at E10.5 and were not recovered after this stage (Fig 1E). The ratios of heterozygous to wildtype offspring suggest no loss of heterozygotes. At three weeks, heterozygotes showed no difference in body weight when compared to wildtypes (Fig 1F). The heterozygous males were fertile and sections from adult testis showed no obvious abnormalities (S4 Fig).

### Trim33 binds active promoters in the testis

**Fig 2:**
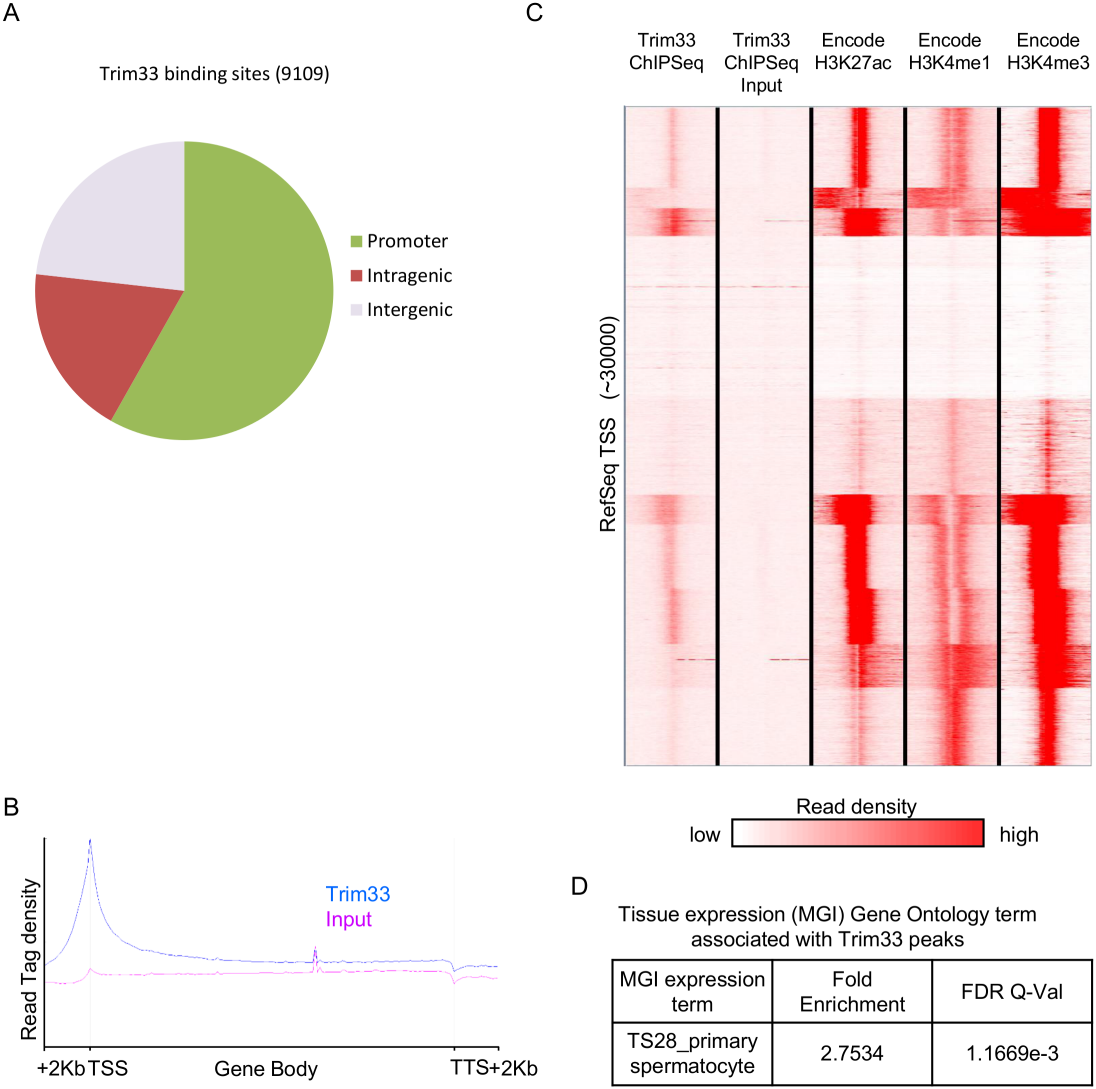
Trim33 binds active promoters in testis. (**A**) ChIP-seq was performed for Trim33 in adult testis. From the 9,109 detected peaks, the percentage that overlap with promoters (2Kb from a TSS), intragenic sequence and intergenic sequence are shown. (**B**) Trim33 occupancy over all RefSeq gene bodies is shown as read tag density along the transcription unit, including 2Kb up and downstream of the transcriptional start and stop site. (**C**) Read density for Trim33 and ENCODE histone marks were calculated across a 6Kb region centred on the 30,000 RefSeq TSSs. Datasets were clustered by read density. (**D**) The GREAT tool was used to test if regions associated with Trim33 peaks were significantly associated with expression of genes in a specific tissue/stage.

To identify the binding sites of Trim33, across the genome, the same anti-Trim33 antibody (Bethyl Laboratories A301-060A) was used to perform ChIP in adult testis followed by high throughput sequencing of Trim33-bound DNA. Approximately 40 million reads were generated for both Trim33 chromatin immunoprecipitated DNA and an Input control and approximately 30 million of these uniquely aligned to the genome in both cases (S1 Table). Peaks were called in the aligned sequence data using the program MACS [14]. This method looks for significant enrichments in the ChIP-seq data file when compared to the Input data file. A total of 9109 peaks were identified across the genome (S2 Table). Trim33 enrichment was validated using ChIP qPCR at all loci tested (four of four) (S5 Fig). The majority of the 9109 peaks occurred within 2Kb from a RefSeq transcription start site (TSS) (Fig 2A). Furthermore, when read density was calculated across the gene body unit for all RefSeq genes, Trim33 was found to be enriched over the transcription start site (Fig 2B).

Using the ENCODE testis dataset, we found that regions of high read density for Trim33 had high read density for H3K4me3 and H3K27ac (Fig 2C). These histone modifications are generally associated with actively transcribed genes and this has been reported specifically in spermatogonial cells [15]. There were a significant proportion of RefSeq genes with both H3K27ac and H3K4me3 that did not have Trim33 ChIP-seq peaks, suggesting that not all genes that are transcriptionally active in the testis are bound by Trim33. Using the GREAT gene ontology tool [16], the 9109 Trim33 peaks were interrogated for association with genes of particular function. The peaks were significantly enriched adjacent to genes that are classified by the MGI expression database as “the TS28_primary spermatocyte” (Fig 2D). This is equivalent to an adult animal’s primary spermatocytes. This was expected, as the majority of Trim33 peaks were located at promoters with active histone marks and likely to be expressed in testis. Primary spermatocytes represent the majority of the cells of the testis.

### Trim33 binds RLTR10B elements in the testis

**Fig 3:**
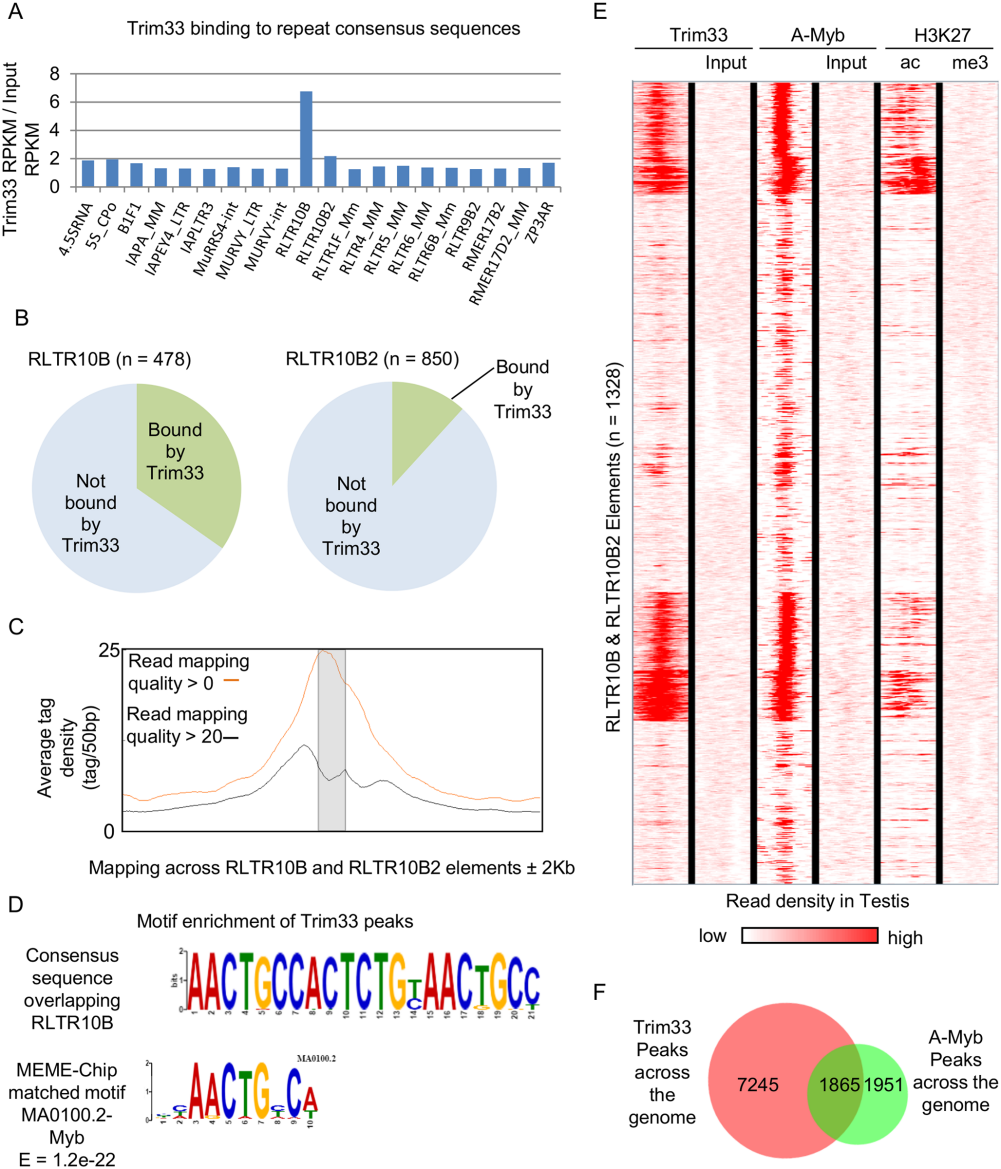
Trim33 binding at RLTR10B and RLTR10B2 elements. (**A**) Trim33 enrichment at retrotransposon classes that show at least a 1.25 fold change over input. RLTR10B and, to a lesser extent RLTR10B2, are enriched in Trim33 binding. (**B**) Trim33 binds to subsets of RLTR10B (166/478) and RLTR10B2 (100/850) repeats in the genome. (**C**) Read density for Trim33 is shown across the RLTR10B and RLTR10B2 element unit (shaded) ± 2Kb. All reads are shown in red and separately all reads with a Bowtie2 read quality of > 20 are shown in black. (**D**) The MEME-chip tool was used to identify enriched motifs in the sequences that overlapped Trim33 binding peaks across the genome in testis. The most significant of these matched a Myb transcription factor consensus sequence within RLTR10B elements. (**E**) Heat plot of ChIP-seq read density, clustered by similarity, for Trim33 and input for all RLTR10B and RLTR10B2 elements, against publically available ChIP-seq data for the A-Myb (plus input), GEO accession GSE44690, in testis and the testis Encode datasets for H3K27ac and H3K27me3. Each element shown is generated from 1Kb either side of the centre of RLTR10Bs and RLTR10B2s in the genome. (**F**) Significant Trim33 ChIP-seq peaks across the genome were compared to significant A-Myb ChIP-seq peaks [18] across the genome, the overlap is shown.

*Trim33* was identified in a screen for genes involved in silencing of a GFP transgene, a locus that had been inserted into the genome. This raised the possibility that it might have a role in regulating other elements recently integrated into the genome, including LINE-elements and LTR-containing retrotransposons. Using the Trim33 ChIP-seq data, we mapped reads to consensus sequences for each repeat annotation from the rodent Repbase repository, which groups elements by sequence, e.g. IAPEZI, IAPLTR1_Mm, IAPLTR2b etc. Of the approximately 1600 repeat consensus sequences, we considered only the 309 that had a RPKM (reads per kilobase per million) >10 in testis (S3 Table). Only 20 classes showed a fold change (over Input) of greater than or equal to 1.25 (Fig 3A and S3 Table). We found that Trim33 was enriched almost seven-fold at RLTR10B and to a lesser extent (two-fold) at RLTR10B2. These are among the youngest retrotransposon elements in the genome [17] and belong to the ERVK family of repetitive elements. Using the RepeatMasker locations of all RLTR10B and RLTR10B2 elements, we found that 35% of the former and 12% of the latter overlapped with Trim33 ChIP-seq peaks (Fig 3B). Some reads were likely to have aligned poorly at individual RLTR10B elements due to their repetitive nature. After filtering out reads with a Bowtie2 mapping quality score of below 20, a large proportion of reads still remained at these sites suggesting that they were uniquely mapped. Importantly, many reads overlapped with non-repeat derived flanking sequences to which short deep sequencing reads are more readily mapped (Fig 3C). Fortuitously, ChIP qPCR validations, discussed in the previous section, included a ChIP-seq peak overlapping a RLTR10B element located in an intron of the gene *Fgf2* (S5 Fig).

### Trim33 binding locations overlap A-Myb ChIP-seq binding sites

Trim33 lacks a classic DNA binding domain, suggesting other mechanisms are required to provide specificity. Studies in other tissues/cell types have found that Trim33 uses either Smad4 or β-catenin to anchor to DNA [8]. To address how Trim33 might be directed to DNA in testis, we used the MEME program to identify any consensus sequences from the Trim33 binding peaks with a high significance, P value < 1.0e-20. These peaks (N = 2338) generally had high enrichment over Input (99% were at least 5 fold enriched) and these were the most likely to represent direct interactions between Trim33 and the transcription factors to which it binds. The three most highly enriched motifs are found in RLTR10B elements, as expected. Using the JASPAR CORE 2014 database of transcription factor DNA-binding sites, a match was found between an RLTR10B MEME-generated consensus sequence and the Myb consensus DNA binding site (Fig 3D). The Myb consensus sequence emerged from 502 individual sites. Several other DNA binding factors were identified with lesser significance, however, no match was found with Smad or β-catenin binding sites (S6 Fig). As ChIP-seq has been carried out for A-Myb (also known as Mybl1) in testis [18], we performed unsupervised hierarchical clustering of ChIP-seq read densities for Trim33, A-Myb and two Encode histone marks, H3K27ac and H3K27me3 (representing active and repressive chromatin, respectively) at RLTR10B elements (Fig 3E). We found good overlap between those RLTR10B elements that bind Trim33, those that bind A-Myb and those enriched for H3K27ac. The repressive chromatin mark H3K27me3 was not enriched at RLTR10B elements. This lack of H3K27me3 enrichment at RLTR10B elements has been reported in ES cells [19]. Given the similar profiles of Trim33 and A-Myb at RLTR10B elements, we compared sites of Trim33 enrichment and those of A-Myb enrichment across the entire genome and found that approximately half of all A-Myb peaks overlapped with Trim33 peaks and approximately one fifth of Trim33 peaks overlap with A-Myb peaks (Fig 3F). These findings suggest that A-Myb has a role in the actions of Trim33 across the genome in testis.

RLTR10B elements were clustered by similarity of the Trim33 and A-Myb read density and those with a high read density for both were found to be enriched for the JASPAR database Myb consensus DNA-binding sequence (S7A Fig). RLTR10B elements that bind Trim33 and A-Myb account for 78% (390/502) of the MYB sites identified in our Trim33 ChIP-seq motif analysis. RLTR10B with no enrichment for Trim33 and A-Myb were not enriched for the Myb DNA-binding sequence (S7A Fig). This supports a role for A-Myb in the function of Trim33 in transcriptional regulation of RLTR10B-containing retrotransposons in the testis. Furthermore, several Trim33 ChIP-seq datasets have recently been carried out in lymphoid cell lines that do not express A-Myb [20]. Reanalysis of these datasets revealed little affinity of Trim33 for RLTR10B elements in these cell types (S7B Fig).

The RLTR10B consensus sequence has four tandem sites matching the JASPAR CORE database Myb consensus binding site located at the 5’ region of the RLTR10B consensus sequence (S8 Fig). Among the broader category of 447 ERV2 elements, 12 contain the consensus Myb binding site but in all cases these are interspersed across the element (S8 Fig).

### TRIM33 regulates RLTR10B elements via ubiquitination of A-MYB

**Fig 4:**
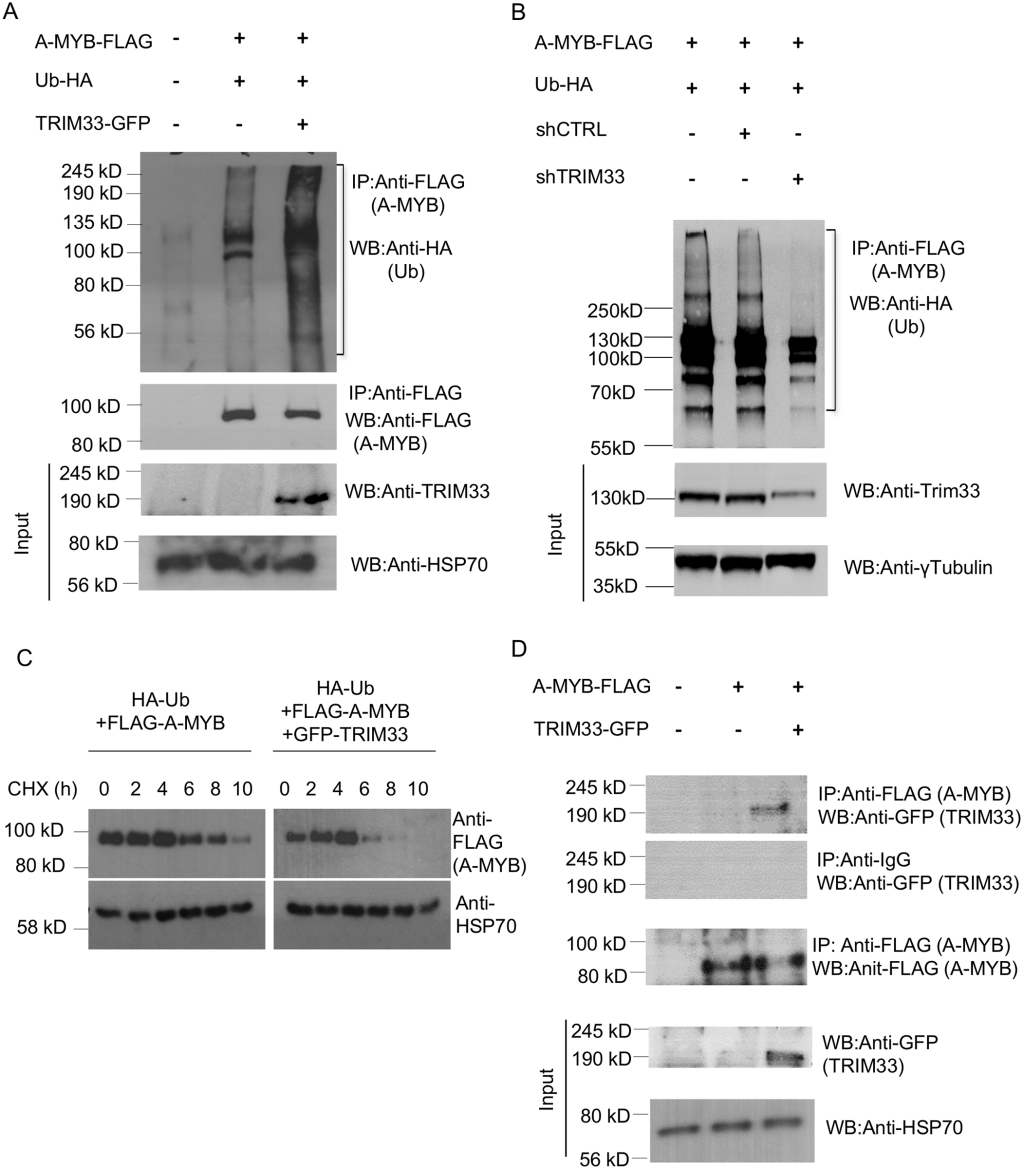
TRIM33 regulates A-MYB stability through ubiquitination. (**A**) HEK293T cells were transfected with HA-tagged ubiquitin and FLAG-tagged A-MYB in the presence or in the absence of GFP-tagged TRIM33. To detect ubiquitinated A-MYB, immunoprecipitation was performed on the lysates using anti-FLAG affinity beads and a Western blot was performed using anti-HA antibodies (top panel). To detect the immunoprecipitated A-MYB, the Western blot was probed with anti-FLAG antibodies (middle panel). Expression of tagged TRIM33 and the loading control HSP70 is shown in the bottom panel. (**B**) HEK293T cells were transfected with HA-tagged ubiquitin and FLAG-tagged A-MYB and either a control or TRIM33 shRNA expression vector. A-MYB was immunoprecipitated and Western blotting was performed as in (**A**) to detect ubiquitinated A-MYB (top panel). Lysates were subjected to Western blotting with anti-TRIM33 antibody to show knock down and anti-γ-Tubulin antibody as a loading control (bottom panels). (**C**) The protein stability of A-MYB in the presence and in the absence of TRIM33 was monitored using the protein translation inhibitor cycloheximide (CHX). Cells were transfected as in (**A**) and CHX was added at 10 μg/ml and samples were analyzed by Western blots at various time points, as shown. HSP70 was used as the loading control. (**D**) FLAG-tagged A-MYB and GFP-tagged TRIM33 were co-expressed in HEK293T cells. Lysates were subjected to immunoprecipitation with anti-FLAG beads or, as a control, anti-IgG beads, and co-immunoprecipitation of TRIM33 with A-MYB was detected by Western blotting with anti-GFP and anti-FLAG antibodies, respectively (top panels). Expression of tagged Trim33 in lysate is shown by Western blotting with anti-GFP antibody and as a loading control anti-HSP70 is also shown (bottom panels).

Given that Trim33 binds to chromatin and functions as a transcriptional repressor via its ubiquitin ligase activity [21], it seemed likely that A-Myb is a testis-specific target of Trim33 ubiquitination. Two ubiquitination sites have been identified on the A-Myb protein, the amino acids K79 and K140 [22]. Combinations of tagged A-MYB, ubiquitin and TRIM33 were overexpressed in HEK293 cells; A-MYB ubiquitination was substantially increased in the presence of TRIM33 (Fig 4A). Knock down of TRIM33 using shRNA decreased the level of ubiquitinated A-MYB (Fig 4B). To demonstrate the functional significance of this, A-MYB levels were monitored in the presence of cycloheximide, which prevents protein translation. In the presence of ubiquitin alone, A-MYB survived to 10 hours whereas in the presence of ubiquitin and TRIM33, the levels of A-MYB survived to only 6 hour time point (Fig 4C). In an attempt to identify an interaction between the co-expressed TRIM33 and A-MYB, we carried out immunoprecipitation for A-MYB (using the FLAG tag) followed by Western blotting for TRIM33 (using the GFP tag). TRIM33 could be detected following immunoprecipitation with anti-FLAG (A-MYB) but not following immunoprecipitation with anti-IgG (Fig 4D). In the absence of a spermatogonial cell line, immortalized MEFs were used to measure the expression of RLTR10B elements following overexpression of A-MYB and ubiquitin. Overexpression of A-MYB resulted in a small increase in RLTR10B expression and this effect was reversed after co-expression with ubiquitin (S9 Fig), consistent with our hypothesis.

### Mice haploinsufficient for Trim33 have altered gene expression in the testis

**Fig 5:**
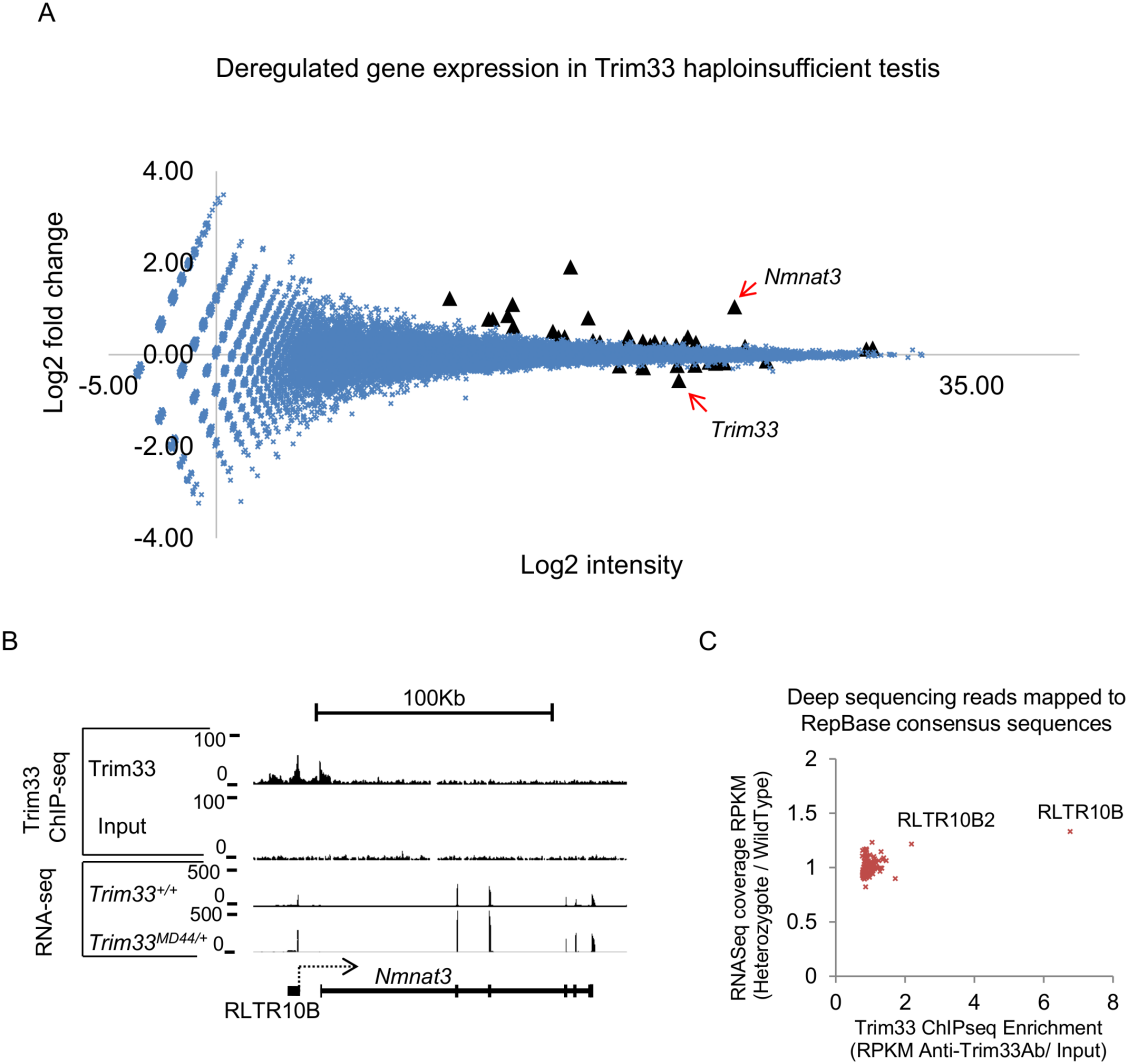
Trim33 regulates a group of genes in the testis. (**A**) The Mean Intensity plot of RNA-seq data shows the log2 fold change of transcripts between wildtype (n = 3) and heterozygotes (n = 4), along a log2 scale of expression intensity (i.e. wildtype levels multiplied by heterozygous levels). Black triangles represent the 39 genes predicted to be significantly differentially expressed between the two sample groups. Trim33 is indicated as significantly lower in heterozygotes. (**B**) Trim33 binding at *Nmnat3*. Visual inspection of the RNA-seq reads suggested that this gene was expressed from an upstream RLTR10B. (**C**) Reads from each individual repeat were mapped to a Repbase consensus sequence. Those repeat classes at which the RPKM was < 10 were excluded. The ratio of RPKM in wildtypes versus that in heterozygotes is shown on the Y-axis. Trim33 ChIP enrichment at each repeat class is shown on the X-axis. Only one class, RLTR10B (indicated), was bound by Trim33 (seven fold enriched) more than threefold above Input values and was at least 1.3 fold upregulated in heterozygous mutant mouse testis.

We were keen to identify loci that might be sensitive to haploinsufficiency of Trim33 in the testis and that overlapped with Trim33 enrichment. We purified RNA from the testis of wildtype (n = 3) and heterozygous *Trim33* ^*MommeD44/+*^ (n = 4) mice and carried out RNA-seq. Using the R package DESeq and Ensembl transcript annotations (NCBI build 37), we identified 39 genes that were significantly differentially expressed (Fig 5A and S4 Table). *Trim33* was ranked third in this list and was 0.68 fold down-regulated, close to expectation. The majority of the changes showed increased expression levels in the mutants, consistent with a role for Trim33 as a repressor of transcription in this tissue. Using RTqPCR we were able to validate the majority of those tested (9 of 11) (S10 Fig). However, only eight of the 39 showed overlap with Trim33 binding peaks. Interestingly, the most significantly deregulated gene, *Nmnat3*, upregulated in the mutants by two-fold, and validated by RTqPCR (S10 Fig) has an upstream RLTR10B element bound by Trim33 (Fig 5B). In the testis, reads for the *Nmnat3* gene start at this element and read across into exon2 (the first coding exon) showing that transcription initiates in the repeat element (S11 Fig). Clonal bisulphite sequencing inside this RLTR10B showed no change in DNA methylation in testis of *MommeD44* heterozygotes compared to that of wildtype mice (S12 Fig), despite a two-fold change in transcription.

We used Cufflinks to establish transcript annotations guided by both the raw RNA-seq data and existing annotations for the mouse mm9 genome to identify other upstream RLTR10Bs that initiate transcription of an adjacent gene. This set was used to identify transcripts that were significantly differentially expressed and linked to a RLTR10B or RLTR10B2. Ten were found (S5 Table), including *Nmnat3*.

A comparison was made between the Trim33 ChIP-seq data mapped against the consensus sequences for each repeat class, described above, and the RNA-seq mapped against the same consensus sequences (Fig 5C). The RLTR10B and RLTR10B2 elements both deviate from all other elements with respect to Trim33 binding, as expected. A slight increase can be seen in expression of the consensus RLTR10B element in heterozygotes compared to wildtypes (fold change = 1.33, p value adjusted for multiple testing = 0.079) (Fig 5C). We did not expect a large increase in expression of this class in the RNA-seq data as the majority of RLTR10B elements do not have A-Myb sites and are not bound by Trim33 (S7A Fig). This increase was validated using RTqPCR with primers designed over the Myb consensus binding sequences but not with primers designed elsewhere in the RLTR10B consensus sequence (S13A Fig), suggesting that the changes seen are limited to those RLTR10B elements with multiple Myb binding sites at the 5’ end. A second individual transcript which was upregulated in heterozygous *MommeD44* mice and overlapping with an RLTR10B element, the *Vmn1r181* gene (S5 Table), was validated using two primers; one annealing upstream of the normal reading frame in the ectopic transcript and the other inside the normal gene reading frame (S13B Fig). RLTR10B expression was measured in E9.5 embryos, in which A-Myb is not expressed. No change was seen across wildtype, heterozygous or homozygous *MommeD44* embryos, consistent with an A-Myb independent function for Trim33 at RLTR10B in whole embryos (S13C Fig).

### Expression of RLTR10Bs in A-Myb deficient mice

Interestingly, *Nmnat3* has been shown to be down regulated ten-fold in the testis of mice with reduced A-Myb function [18]. Using their raw RNA-seq data and the consensus RLTR10B sequence, expression of RLTR10Bs was found to be decreased two-fold in mice heterozygous for a mutation in *A-Myb* and ten-fold in mice homozygous for the mutation (S14 Fig). These findings are consistent with a central role for A-Myb in transcriptional activity of the RLTR10B elements.

## Discussion

### Silencing of transposable elements in mammals

Transposable elements have been a driving force in the structure and evolution of the mammalian genome. Deep sequencing of 17 mouse genomes has revealed over 100,000 transposable element variants that have survived selection over the past 2 million years of *Mus* lineage evolution [23]. From an inferred evolutionary history of TE family activity, ERVs and in particular ERVKs, appear to be expanding rapidly in the mouse [24]. The ERVK class, also known as ERV2, contains IAPs, Etn/MusD and RLTR10B-containing retrotransposons such as MMERVK10C. The vast majority of the expansion of LTR retrotransposons is thought to have occurred in the paternal germline [23]. This is consistent with findings from a study in which IAP-GFP transgenes were inserted into the mouse genome. Expression of these transgenes was found to be limited to the male germline [25]. Most studies that have investigated the transcriptional regulation of the ERV2 class of repeats have focussed on piRNA pathways [23]. Directed mutagenesis in mice has identified nine proteins with a key role in protecting the male germline against retrotransposition of IAPs, Etn/MusD and MMERVK10C elements; Dnmt3L, Dnmt3a, Miwi2, Mili, Mael, Tdrd1, Tdrd9, Gasz, Tex19.1 and most function in the piRNA pathway [23]. Several groups have reported on factors that silence MMERVK10Cs in the germline [26, 27] but the mechanisms of silencing of RLTR10Bs has not been understood prior to our studies. Here we describe the first factor involved in silencing of the RLTR10Bs elements.

### Trim33 is a transcriptional corepressor with important roles in development and cancer

*Trim33* has been identified in a number of screens designed to find genes involved in transcriptional silencing. It was found in a RNA inhibition (RNAi) screen carried out in a human cell line to find proteins required for transgene silencing [28]. The fly homologue of *Trim33*, *Bonus*, was identified as a suppressor of variegation (i.e. reduced levels of Bonus result in an increased proportion of cells expressing the reporter transgene) when tested for its ability to alter transcription of a variegating reporter locus [29]. Here we report, the identification of *Trim33* in a mutagenesis screen for modifiers of transcriptional silencing in the mouse. In this screen it also behaves as a suppressor of variegation.

Trim33 was identified in a zebrafish screen for genes involved in the development of the haematopoietic system [30, 31]. It is known to be required for development of ectoderm in Xenopus [32]. Knockout of Trim33 in the mouse has been shown to result in embryonic lethality as a consequence of excessive TGFβ/Nodal signalling [33]. Our findings of embryonic lethality of embryos homozygous for the *Trim* ^*Mommed44*^ allele are consistent with this report.

Trim33 is not thought to bind DNA directly and available evidence suggests that its transcriptional effects occur via DNA-binding cofactors. For example, the role of Trim33 in TGF-beta signalling in cell lines has been shown to involve the Smad transcription factors that bind DNA in a sequence-specific manner [8, 34]. [It has been suggested that the mechanism by which Trim33 inhibits TGF-beta signalling during the development of ectoderm in Xenopus involves the ubiquitination of Smad4 [32]. The model proposes that Trim33 inhibits expression of the target locus by destabilising the Smad cofactor.]

The role of the chromatin binding domains (PHD and Bromodomain) of Trim33 remains unclear. Previous studies, following overexpression of the chromatin binding domains in somatic cell lines, mapped the binding specificity to H3K4me0 and acetylated lysine residues [8, 21]. However, our ChIP-seq studies, carried out *in vivo*, found that most Trim33 peaks in testis overlap with peaks for H3K4me3 and H3K27ac and presumably not for H3K4me0. Others have suggested that the PHD and Bromodomain of Trim33 are required to activate its ubiquitin ligase activity [21].

Multiple mouse models of cancer have demonstrated a role for Trim33 as a tumour suppressor [11, 35, 36]. Trim33 was found to act as a tumour suppressor in the pancreas of mice and humans [35]. The mechanism underlying the tumour suppressor function is thought to be Smad4-independent [37]. Recent studies have found that TRIM33 abolishes tumour cell proliferation and tumorigenesis by degrading nuclear β-catenin via ubiquitination [36].

Taken together, these findings indicate Trim33 can be considered a corepressor of transcription that functions by ubiquitinating DNA-binding cofactors and that it has important roles in development and cancer.

### Trim33 silences a specific class of young active retrotransposons in testis

Trim33 is expressed an order of magnitude higher in the testis than in any other adult mouse tissue [13]. It is expressed in spermatogonia, preleptotene spermatocytes and round spermatids but its function in these cells is unknown. It is also expressed in one class of the somatic cells in the testis, the Sertoli cells, but these are much less abundant than the germ cells. Since it has been identified in a number of screens for gene silencing and since the silencing of retrotransposons is a critical function of the germline, we hypothesised that it might have a role in this process.

Our study is the first report of ChIP-seq for Trim33 in any tissue. We show that Trim33 binds many active gene promoters in the testis (i.e. those promoters marked by H3K4me3 and H3K27ac). Trim33 also shows enrichment at RLTR10B elements, a subgroup of ERVK LTR retrotransposons, consistent with our hypothesis. RLTR10Bs are among the chronologically youngest retrotransposable elements in the mouse genome, ranked 5^th^ out of 546 elements analysed [17]. The only groups considered younger than RLTR10Bs are IAPs and these are known to have retrotranspositional activity in the mouse [38]. In general, the youngest retrotransposable elements will have accumulated less defects (i.e. mutation of DNA binding sites) and will have retained the ability to be expressed.

Knowing that haploinsufficiency for Trim33 was sufficiently disruptive to increase expression of the reporter transgene in our mutagenesis screen, we were keen to test whether it would alter the expression of other genes in the testis. RNA-seq suggests no dramatic effect across the genome but confirms a role for Trim33 in silencing RLTR10B. The fact that the transgene and the RLTR10B elements, but not other Trim33-bound loci, are sensitive to Trim33 dosage is not understood. It might be that other Trim family members (and other factors) can compensate for reduced levels of Trim33 at most loci. It is likely that in the complete absence of Trim33, many of the Trim33 bound loci would be affected but the early demise of the homozygous embryo precludes testing.

### Trim33 is likely to act via A-Myb

We have identified a role for Trim33 in binding and silencing the RLTR10B-containing class of retrotransposon in testis. Many other groups of retrotransposons, including active IAP elements, are enriched for the same histone marks found on RLTR10B elements [19] but are not bound by Trim33, suggesting that Trim33 does not bind via its bromodomain or PHD finger. On the assumption that Trim33 binds DNA via a transcription factor, we searched for a transcription factor consensus binding site and found four separate Myb binding sites at the 5’ end of the RLTR10B consensus sequence and the individual RLTR10B upstream of *Nmnat3*. This tandem arrangement has previously been shown to increase the affinity for Myb binding to DNA [39] A-Myb is a master regulator of male meiosis and is expressed specifically during spermatogenesis [40] but has not previously been reported to bind retrotransposons. Given the overlapping ChIP-seq signal between A-Myb and Trim33 at these repeats (Fig 3E), it is likely that Trim33 functions to repress transcription at these sites via A-Myb.

The *Momme* screen reporter transgene contains an AACTGTCT element in the HS40 alpha globin hypersensitive site and this fits the MYB binding consensus site–AACTG(C/T)C(A/T). A-MYB has been shown to act as a transcriptional activator in peripheral blood cells [41]. It is reasonable to suggest that Trim33 acts as a transcriptional repressor of the reporter transgene via ubiquitination of A-Myb (or another target of Trim33 ubiquitination), although we have no direct evidence of this.

Myb family members are subject to several types of post-translational modifications, including phosphorylation, acetylation, and ubiquitination. Furthermore, the ubiquitination of B-Myb and C-Myb has been shown to inhibit the transcriptional activation functions of these two factors [42, 43]. Trim28, like Trim33, can ubiquitinate proteins and has been shown to bind to C-Myb and repress its ability to function as a transcriptional activator [44]. Here, we have demonstrated that Trim33 can ubiquitinate A-Myb and that this regulates the abundance of A-Myb, probably by the ubiquitination–proteasome pathway as has been suggested for Trim33 mediated degradation of β-catenin [36].

**Fig 6:**
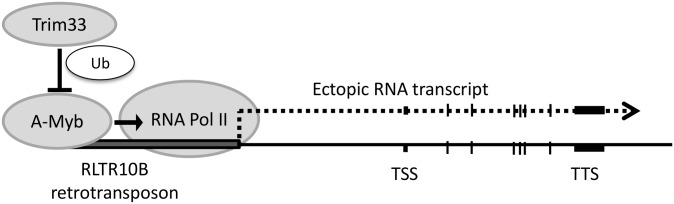
Model of Trim33 function at active RLTR10B retrotransposons in the testis. An RLTR10B element that acts as an alternative promoter of a nearby gene is shown. A-Myb recruits RNA Pol II and drives transcription. Trim33, acting as an E3 ubiquitin ligase, ubiquitinates and destabilises A-Myb, resulting in decreased expression of the locus.

Publically available datasets show that reduction in A-Myb in mouse testis results in a decrease in expression of *Nmnat3* [18]. We have reanalysed their datasets to search for effects on repeats and find dramatically decreased expression of RLTR10Bs (S14 Fig). In addition, the cell types in the adult testis that specifically express high levels of *Trim33* overlap with those that express high levels of *A-Myb* [40]. Given that Trim33 can ubiquitinate and regulate the abundance of A-Myb, a simple model of the mechanism by which Trim33 silences RLTR10B elements in the germline would include the ubiquitination of A-Myb (Fig 6).

Transcriptional activity at retrotransposons is species specific, consistent with rapid evolution of retroviral subtypes [23]. It is likely that a subset of RLTR10Bs have recently evolved Myb DNA binding sites to capitalise on the critical role that the A-Myb transcription factor has in gene expression in germ cells in order to ensure their continued retrotransposition. Suppression of A-Myb by Trim33 provides a plausible mechanism by which the host keeps retrotransposition in check.

## Materials and Methods

### Mouse strains

The ENU screen was carried out in *Line3*, a transgenic line that is homozygous for the GFP transgene and on an FVB/NJ inbred background, as previously described [4]. Maintenance of the *MommeD44* allele was carried out on the *Line3* background. The *Line3C* was used for linkage studies and was produced by crossing *Line3* to C57BL/6J for 10 generations and selecting for homozygosity of the GFP transgene. All breeding crosses and experimental procedures using the *MommeD44* allele were carried out using mice at least 5 generations from the founder mouse. Procedures were approved by the Animal Ethics Committee of LaTrobe University, under approval numbers AEC 12–74 and AEC 12–75. All mice sacrificed in the study were anesthetized using isoflurane and euthanised by cervical dislocation.

### Plasmids and shRNAs

The expression vector encoding A-MYB-FLAG was purchased from OriGene (Rockville, MD, USA). TRIM33-GFP was obtained from DR Kyle Miller (Addgene plasmid #65399). Knockdown shRNA for Trim33 were obtained from Dr Joan Massague (Addgene plasmid # 15728).

### Genotyping PCR

PCR was carried out using DNA from tail tips using the following conditions: 94 degrees Celsius 6 minutes, 30x cycles with 94 degrees Celsius 30 seconds, 60 degrees Celsius 45 seconds 72 degrees Celsius 45 seconds, followed by a final annealing step of 72 degrees Celsius 6 minutes. All primers used in the study are described in S6 Table.

### Flow cytometry

Flow cytometry of blood from 3 week old mice collected in FACSFlow Sheath Fluid (BD Biosciences) was carried out and analysed on a Guava easyCyte HT (Merck/Millipore, Darmstadt, Germany) and with the Guava InCyte software, respectively. Erythrocyte green fluorescence (525nm) was recorded and a GFP-positive gate was set to exclude 99% of wildtype erythrocytes.

### Linkage analysis

*MommeD44* heterozygous mice were backcrossed twice to *Line3C* (see above) and phenotyped for GFP expression by flow cytometry. DNA from tail tissue collected during flow cytometry procedures was used to perform linkage analysis. The Illumina GoldenGate genotyping assay (Mouse Medium Density Linkage Panel) was used with 10 wildtype and 13 heterozygous mice. *MommeD44* wildtype samples should only have heterozygous C57BL/6J SNPs surrounding the causative mutation and *MommeD44* mutants should have FVB and C57BL/6J SNPs at this interval. The Mouse Medium Density Linkage panel contains 766 measurable SNPs between C57BL/6J and FVB/NJ. Samples were genotyped following the Illumina protocol and genotype calls were made using the Genotyping module of the GenomeStudio v1.1 software. Only samples with a call rate >95 were accepted. The linked interval was identified based on a peak in the LOD score. Fine mapping was carried out using primers amplifying C57BL/6J or FVB SNP loci that could be cut with restriction enzymes to determine genotype. Of the 95 F2 backcrosses, 8 mice had SNP profiles that were inconsistent with the mapping and were excluded. This small (<10%) error rate in phenotyping is commonly encountered in this screen [4].

### Whole exome deep sequencing and SNP calling

Exome capture was performed using the Roche NimbleGen reagents (SeqCap EZ Mouse Exome, version Beta 2, 110603_MM9_exome_rebal_2EZ_HX1, Madison, WI, USA) as per the Illumina optimized Roche NimbleGen SeqCap User’s Guide (version 1.0) and using a Bioruptor (Diagenode, Liège, Belgium) for DNA fragmentation. Libraries were sequenced using the illumina GAIIx platform and reads were aligned to the mouse (build 37, mm9) genome using bwa aln and bwa sampe programs [45]. For further details see [3].

As control sequence, that lacked *MommeD44* ENU mutations, other *MommeD* lines previously identified in the screen and described elsewhere were used [3]. Varscan output was scanned manually for likely heterozygous mutations that could be validated in additional *MommeD44* mutant mice. Sanger Sequencing or restriction enzyme digest with the enzyme Mse1 (New England Biolabs) that specifically cuts at the ENU mutation site in the *Trim33* ^*MommeD44*^ allele was used.

### Embryo dissections

Timed matings between heterozygous mutant females and heterozygous mutant males were set up and the detection of a vaginal plug was counted as 0.5 dpc. Genotyping was carried out using DNA extracted from embryos.

### Bisulphite sequencing of the RLTR10B upstream of *Nmnat3*

Bisulphite conversion was carried out on 1ug of DNA extracted from whole testis using the EpiTect Bisulphite Kit (Qiagen, Doncaster, VIC, Australia) according to the manufacturer’s instructions. The bisulphite conversion rate was at least 99% and sequences were analysed using the BiQ Analyser software. Oligonucleotides are provided in S6 Table. PCR cycling conditions were as follows: 95 degrees Celsius 10 minutes, 30x cycles with 95 degrees Celsius 15 seconds 55 degrees Celsius 15 seconds, 72 degrees Celsius 30 seconds. PCR products were cloned using a pGEM-T Easy Vector (Promega, Alexandria, NSW, Australia) and sequenced using The BigDye Terminator v3.1 Cycle Sequencing Kit (Life technologies, Mulgrave, Victoria, Australia) as per kit instructions.

### Cell culture, transfection and RNAi

HEK293T and mouse embryonic fibroblasts were cultured in DMEM supplemented with 10% fetal calf serum (Invitrogen, Scoresby, VIC, Australia) at 37ᴼC in a humidified 10% CO2 incubator. For transient expression of cDNA or RNAi vectors, HEK293 or MEF cells were transfected with expression plasmids using the Fugene 6 reagent (11814443001, Roche, Castle Hill, NSW, Australia) following the manufacturer’s instructions. For A-MYB survival assays, cells were transfected with indicated plasmids for 48 hours and then treated with cycloheximide to a final concentration of 10 ug/ml. Cells were lysed at indicated time points and A-MYB protein levels were assessed by Western blotting.

### Protein extraction, immunoprecipitations and western blotting

Whole-cell extract was prepared from testis of adult (12 week old) mice in 8M urea lysis reducing buffer (8 M urea, 1/10 vol. glycerol, 1/20 vol. 20% SDS, 1/2,000 vol. 1 M dithiothreitol, 1/100 vol. 1 M Tris, pH 6.8). For ubiquitination assays, HEK293T cells were lysed in RIPA buffer (Tris 50mM, NaCl 150mM, NP-40 1%, DOC 0.5%, SDS 0.1%), diluted 1:10 in ONYX buffer (Tris 20mM, NaCL 135mM, MgCl_2_ 1.5mM, EGTA 1mM, Triton X-100 1%, Glycerol 10%) and incubated overnight with anti-FLAG antibody at 4ᴼC. Following incubation, antibodies were washed in ONYX buffer and proteins were eluted in 8M urea lysis buffer. For protein co-immunoprecipitations the Nuclear Complex Co-IP Kit (54001, Active Motif, Carlsbad, CA, USA) was used as per manufacturer’s instructions. BCA (Thermo Scientific, Waltham, MA, USA) was used to quantify protein and total lysates were separated according to size on polyacrylamide gels (Biorad, Gladesville, New South Wales, Australia).

Antibodies used for western blotting were rabbit polyclonal anti-Trim33 (A301-060A, Bethyl Laboratories, Montgomery, TX, USA), rabbit polyclonal anti-A-Myb (HPA008791, Sigma Aldrich, Castle Hill, NSW, Australia), mouse monoclonal anti-GFP (11814460001, Roche, Castle Hill, NSW, Australia), mouse monoclonal anti-HA (6E2, Cell Signaling, Boston, MA, USA), mouse monoclonal anti-Gapdh (MAB374, Merck/Millipore, Darmstadt, Germany) mouse monoclonal anti-HSP70 (MA3-028, Scoresby VIC, Australia) and rabbit polyclonal anti-γ-Tubulin (T5192, Sigma Aldrich, Castle Hill, NSW, Australia). Anti-Flag was a gift from Dr Lorraine O'Reilly at the Walter and Eliza Hall Institute of Medical Research. Antibodies used for immunoprecipitation were bead-conjugated anti-FLAG (M8823, Sigma Aldrich, Castle Hill, NSW, Australia) and anti-IgG (sc-2345, Santa Cruz Biotechnology, Dallas, TX, USA).

### RNA isolation, cDNA conversion and reverse transcriptase quantitative PCR (RTqPCR)

Total RNA was extracted from snap frozen tissues or cells using TRIzole reagent (Life technologies, Mulgrave, Victoria, Australia) according to manufacturer instructions. cDNA synthesis was carried out from total RNA using the QuantiTect Reverse Transcription Kit (Qiagen, Doncaster, VIC, Australia) and RTqPCR was performed with the QuantiTect SYBR Green reagent (Qiagen, Doncaster, VIC, Australia) with primers designed to span exon junctions in mRNA (S6 Table). Samples were run on the CFX384 Touch Real-Time PCR Detection System (Biorad, Gladesville, New South Wales, Australia), with the following conditions: 95 degrees Celsius 10 minutes, 39x cycles with 95 degrees Celsius 15 seconds then 60 degrees Celsius 1 minute, with a final step of 95 degrees Celsius 15 seconds. Each experimental sample consisted of three technical replicates and reverse transcriptase negative samples; a melt curve analysis was carried out after each run to confirm unique PCR product amplification. Relative cDNA abundance was calculated using the delta delta CT method normalizing to housekeeper gene expression indicated in the figures. Statistical analysis was performed using Student’s *t* test.

### RNA-seq

RNA sequencing was carried out from total RNA submitted to the Australia Genome Research Facility (AGRF, Parkville, Victoria, Australia). At least 20 million 100bp single end reads were generated on an Illumina HiSeq platform for each sample, using libraries generated using the illumina TruSeq RNA Sample Preparation kit (Illumina, San Diego, CA, USA). Initial QC was performed by AGRF. Reads were aligned to the mouse genome (NCBI 37, mm9) using the program Tophat (version 2.0.11) [46] with the following parameters: -I 100000—library-type = fr-unstranded—read-edit-dist 3—no-coverage-search—read-mismatches 3. Read counts for gene exons were extracted using the program htseq-count (version 0.6.1) [47] with the options -s no -m intersection-strict and using gene annotations from Ensembl (release 67). Differential gene expression was assessed using the R-package DEseq [48], with default parameters. Genes were considered differentially expressed when an adjusted p value of at least 0.05. Where indicated in the text, the program Cufflinks (version 2.2.1) [49] was used to estimate differential gene expression of transcripts by creating annotations based on mapped reads. Mapped RNA-seq reads (above) were used to create transcript annotations using default settings for each sample, these were merged and then used to estimated differential expression of transcripts.

### ChIP-seq

Testis tissue from three 12 week old wildtype mice were snap-frozen and sent to Active Motif for ChIP, library preparation, sequencing and initial data analysis. The rabbit polyclonal anti-Trim33 (A301-060A, Bethyl Laboratories, Montgomery, TX, USA) was used for ChIP. Sequencing was carried out for 75mer read lengths on the NextSeq 500 platform (Illumina, San Diego, CA, USA). Reads were aligned using the BWA program [45] with default settings. Peak calling was done with MACS (Version 1.4.2) [14] by first filtering out duplicate reads and reads with a mapping quality of less than 25, then using default parameters and the following options -s 75—bw 200 -m 10 30 –p 0.0000001 (S1 Table). All heat plot and read tag density figures were generated using the seqMiner program (version 1.3.3) [50] using default parameters and ChIP-seq data aligned to the mouse genome (NCBI 37, mm9) with the program Bowtie2 (version 2.2.2) [51] with default settings. Heat plots were generated by subsampling all datasets to approximately 16 million reads. In the case of publically available ENCODE datasets, aligned reads in bam format were downloaded and were subsampled to approximately 16 million reads or in the case of H3K27ac, two biological replicates were combined to generate a data file with 16 million reads. The data sets supporting the results of this article are available in the Gene Expression Omnibus (GEO) repository, [GSE68617]. Motif discovery and enrichment was performed with highly significant Trim33 peaks (P value <1.0d-20, region summit +- 650bp) using the MEME-ChIP (version 4.10.0) [52] and MEME suite programs MEME, DREAME, CentriMo and Tomtom with default settings. Gene ontology analysis was carried out using Trim33 peak locations across the genome with the GREAT tool (version 2.0) [16] with default settings.

### Mapping to repeat elements

To estimate enrichment of repeat elements for ChIP-seq and RNA-seq datasets reads were mapped to a repeat assembly file containing a single FASTA entry for each repeat type defined in the rodent repeat sequences RepBase database [53] (update 20.02). The Bowtie2 (version 2.2.2) [51] aligner was used to map reads aligning to each FASTA entry using default settings and RPKM values were extracted from the number of reads aligned at each entry and the library size for each data file.

### Availability of supporting data

The data sets supporting the results of this article are available in the NCBI Gene Expression Omnibus under the accession code GSE68617.

## Supporting Information

S1 Fig Linkage analysis. Single Nucleotide Polymorphism mapping using the Illumina Golden Gate SNP Chip assay indicated a peak in LOD score between 75 and 155Mb on chromosome 3.
(PDF)

S2 Fig GFP expressing in MommeD44 mice. Mice from the *MommeD44* colony (n = 113) were measured for their percentage of GFP expressing erythrocytes and grouped by genotype for the *Trim33* mutation.
(PDF)

S3 Fig Loss of Trim33 in *MommeD44* heterozygous and homozygous mice. **(A)** The Trim33 antibody specificity at a 1:10000 dilution was tested using protein extract from three independent wildtype samples. The lysate was separated on a polyacrylamide gel. **(B)** Wildtype (n = 7) and heterozygote (n = 6) testis extracts incubated with the Trim33 antibody and an antibody against γ Tubulin. **(C)** Densitometry analysis of **B** shows Trim33 is significantly reduced in heterozygotes compared to wildtypes (p value < 0.05), error bars indicate SEM. **(D)** Heterozygote and homozygote protein extracts, from pooled E9.5 embryos (N = 4 per sample), incubated with the Trim33 antibody and antibodies directed against the housekeepers γ Tubulin and Gapdh.
(PDF)

S4 Fig Histology sections from *MommeD44* mouse testis. Mice heterozygous for *MommeD44* show no obvious differences in histological sections after staining with haematoxylin, compared to *MommeD44* wildtype mice.
(PDF)

S5 Fig ChIP-seq validation at Trim33-bound loci. **(A)** Screenshots of Trim33 ChIP-seq (and Input) read density for four sites that were identified as Trim33 binding peaks. Shown are the 5kb loci centred on each of the peaks and labelled according to their distance from the nearest gene transcription start site: an intronic locus located within the *Fgf2* gene, two gene promoter loci (*Ccdc28a* + *Pdzd8*) and an intergenic Trim33 binding site 5Kb upstream of the gene *Heyl*. The intronic locus within *Fgf2* gene is located at an RLTR10B retrotransposon. **(B)** ChIP qPCR for six loci, two representing negative controls (Untr6 + Untr17) with primers located in regions not enriched for Trim33 and the four Trim33 enriched regions from **A**. Error bars indicate S.D. from 3 technical replicates.
(PDF)

S6 Fig Motifs identified by MEME. Several motifs were significantly enriched in highly significant (P value < 1.0e-20) Trim33 ChIP-seq peaks (N = 2338) that were significantly homologous to known DNA binding consensus sequences from the JASPAR CORE 2014 database
(PDF)

S7 Fig Trim33 and binding is dependent on A-Myb at RLTR10B elements. (**A**) Heat plot of ChIP-seq read density, clustered by similarity, for publically available ChIP-seq data for the A-Myb (plus input)–GEO accession GSE44690 and Trim33 and input in testis, at all RLTR10B and RLTR10B2 elements. Of the two clusters, only cluster 1 is enriched in the Jaspar Core Database Myb consensus binding sequence using the MEME program. (**B**) ChIP-seq data from another study [20], accession number GSE66233, was mapped to the RLTR10B RepBase consensus sequence. Enrichment values are from one ChIP-seq experiment per cell line as the fraction of the RPKM supporting binding compared to the input RPKM. Shown also is the ChIP-seq data from testis mapped to the RLTR10B element.
(PDF)

S8 Fig Myb motif enrichment at ERVK elements. The MEME suite program MAST was used to search for Jaspar Core Database Myb consensus binding sites (shown in green) in the 447 ERV2 elements in the rodent RepBase database. Those elements with an E value of less than 10 are shown. The E value for each element is equal to the combined p-value of the sequence times the number of sequences searched.
(PDF)

S9 Fig Ubiquitination of A-Myb reduces expression of RLTR10B elements. Immortalized MEF cells were transfected with FLAG-tagged A-MYB or FLAG-tagged A-MYB and HA-tagged ubiquitin for 36h. RLTR10B retrotransposon expression was measured and normalized to the expression of housekeeper genes *Hprt* and *Gapdh*. RLTR10B expression increased when A-MYB was overexpressed and the effect is reversed when ubiquitin was also overexpressed. Error bars indicate SEM, * p-value > 0.05. The expression levels of Flag-A-MYB in MEFs and HSP70 are shown (bottom panel).
(PDF)

S10 Fig Testis RNA-seq data validation using RTqPCR. The expression for genes predicted to be significantly differentially regulated by RNA-seq (Fig 5) was tested by RTqPCR. Genes that displayed high expression in testis (greater than 100 read alignments) and fold change of at least 1.4X were chosen. Expression of the indicated genes was measured using primers to the transcript (across exon junctions) and normalized to two housekeeping genes, *Hprt* and *Rps2*, the number of samples for each group was at least 4, * p value ≤ 0.05, error bars = SEM. *Ptgds* and *Serpina3n* did not validate as significantly different between wildtypes and heterozygotes.
(PDF)

S11 Fig Splicing from an upstream RLTR10B element into the *Nmnat3* gene. Splice junction tracks generated by the IGV genome browser program are shown. Each of the tracks represents a mRNA sequence built by the IGV browser program from the split reads mapped by the testis RNA-seq data. The thickness of the bands indicates the relative coverage of split reads supporting usage of a particular splice site. The majority of mRNA across the *Nmnat3* locus begins at the RLTR10B element upstream.
(PDF)

S12 Fig DNA methylation at a differentially expressed RLTR10B element in *MommeD44* heterozygous mice. DNA methylation was measured inside the RLTR10B that functions as an upstream promoter of the *Nmnat3* gene in testis. Bisulfite primer design inside the locus is indicated. The methylation state of individual animals (two per genotype) is shown as columns of cloned PCR products. Filled in circles represent methylated CpGs and white circles represent unmethylated CpGs. Each mouse is represented by eight or greater clones and averages for each sample and the two groups are indicated. The bisulfite conversion rate of the non CpG cytosines is indicated as 99%.
(PDF)

S13 Fig RTqPCR validation of RLTR10B. (**A**) Expression of RLTR10B elements in testis was measured using primer pairs that either included the Myb binding sites (F2 –R1) or not (F1 –R1). Increased expression of RLTR10B elements containing the Myb binding sites was seen in *MommeD44* heterozygotes. Expression was normalized to two housekeeping genes, *Hprt* and *Rps2*, and then normalised to the average expression levels from wildtype mice. The number of samples for each group was at least 3, * p value ≤ 0.05, error bars = SEM. (**B**) Expression of an ectopic transcript, originating at an RLTR10B upstream of the *Vmn1r181* gene, was measured in *MommeD44* heterozygous and wildtype testis. Primer annealing locations are shown. Increased expression was detected in heterozygous tissue, normalized to the housekeeping genes *Hprt* and *Rps2*, * p value ≤ 0.05, error bars = SEM. (**C**) Expression of RLTR10B elements was measured in E9.5 embryos from the same litter; wildtype, heterozygous or homozygous for the *MommeD44* mutation. No change in expression was detected across genotypes, normalized to the housekeeping gene *Gapdh*.
(PDF)

S14 Fig RLTR10B expression in testis of mice with decreased A-Myb activity. RNA-seq data from another study [18], accession number GSE44690, was mapped to the RLTR10B RepBase consensus sequence. RPKM values are from one biological replicate per genotype for each time point.
(PDF)

S1 Table ChIP-seq run statistics. (XLSX)

S2 Table Trim33 ChIP-seq binding peaks (NCBI build 37) identified in pooled wildtype testis (n = 3 mice). Bowtie2 was used to map raw data from Trim33 ChIP-seq to consensus sequences from the rodent RepBase repository. RPKM values were used to normalize for the size of each element and the library size
(XLSX)

S3 Table Trim33 binding to repeat consensus sequences. Bowtie2 was used to map raw data from Trim33 ChIP-seq to consensus sequences from the rodent RepBase repository. RPKM values were used to normalize for the size of each element and the library size
(XLSX)

S4 Table Genes differentially expressed in MommeD44 heterozygous mice (n = 4) compared to wildtype mice (n = 3). RNA-seq data was mapped back to the Ensembl annotated (NCBI build 37) genome and DEseq was used to identify differentially expressed genes.
(XLSX)

S5 Table Differentially expressed transcripts (between heterozygous and wildtype MommeD44 mice) overlapping with RLTR10B or RLTR10B2 elements. A set of transcript annotations was created using Cufflinks and the mapped RNA-seq data. 10 overlaps were identified.
(XLSX)

S6 Table Primer sequences. (XLSX)
