# Supplementary figures and images for: Trim33 Binds and Silences a Class of Young Endogenous Retroviruses in the Mouse Testis; a Novel Component of the Arms Race between Retrotransposons and the Host Genome
Source: PLoS Genet. 2015 Dec 1;11(12):e1005693. doi: 10.1371/journal.pgen.1005693 (PMC4666613; doi:10.1371/journal.pgen.1005693)

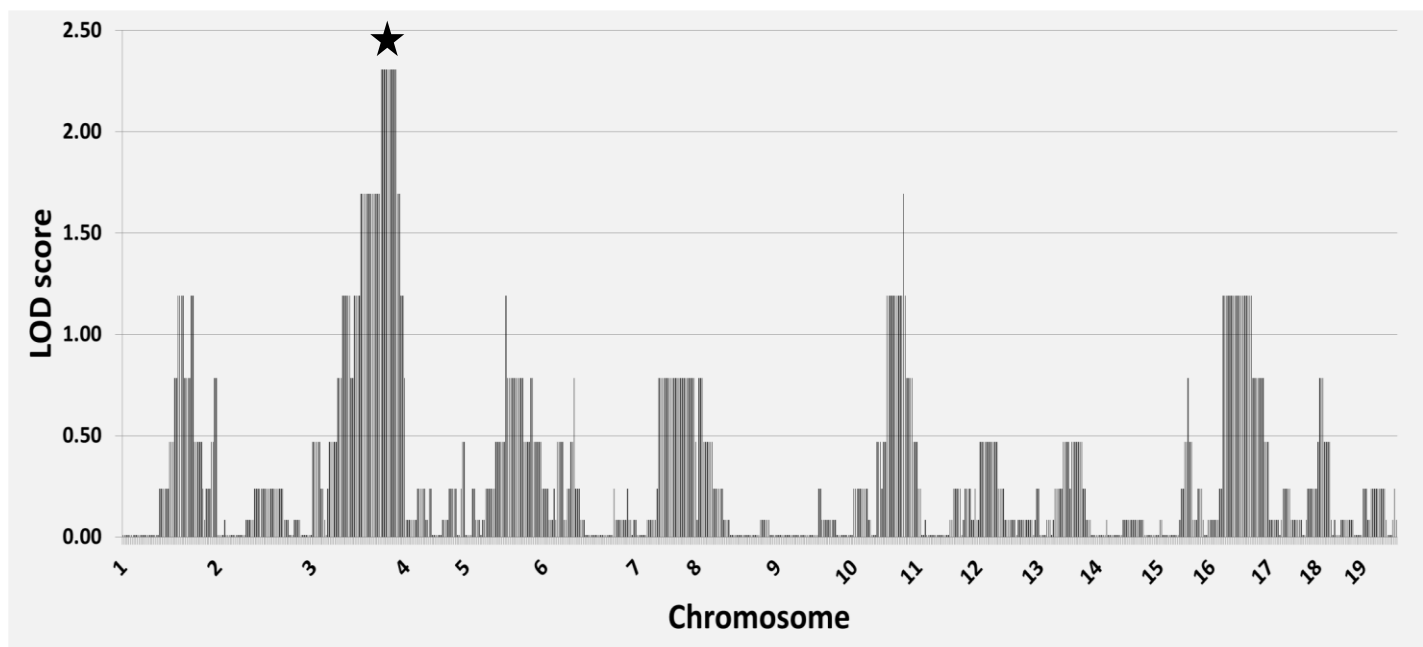

Supplement: S1 Fig — Single Nucleotide Polymorphism mapping using the Illumina Golden Gate SNP Chip assay indicated a peak in LOD score between 75 and 155Mb on chromosome 3. (PDF) [file pgen.1005693.s001.pdf]

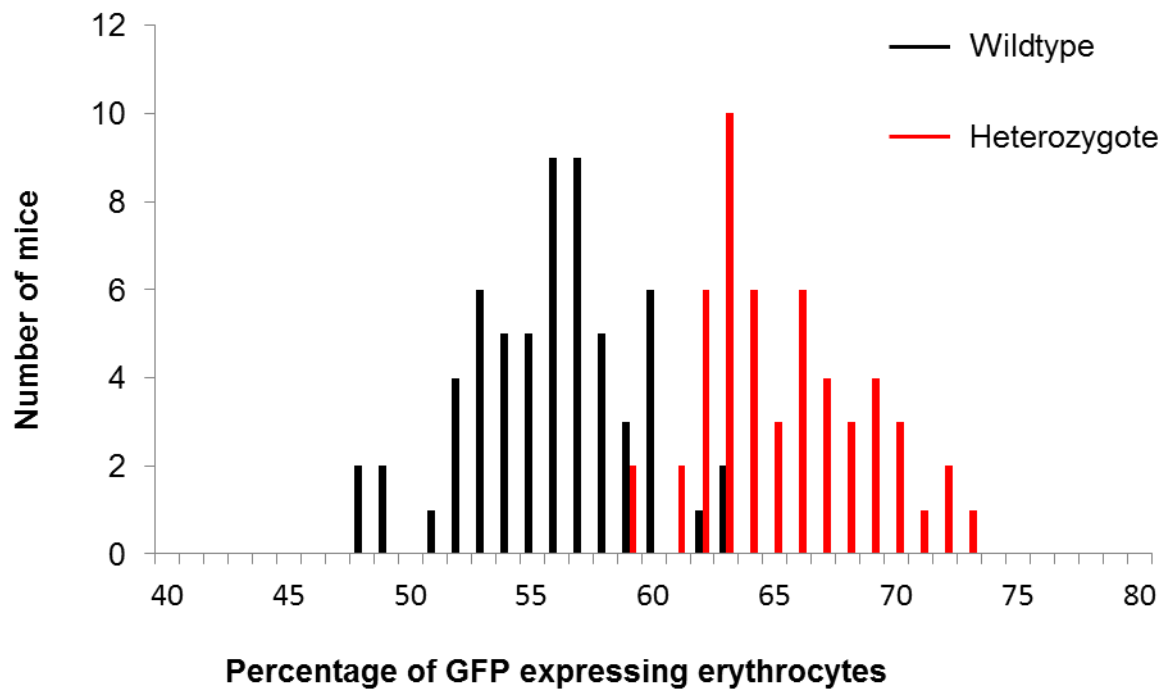

Supplement: S2 Fig — Mice from the MommeD44 colony (n = 113) were measured for their percentage of GFP expressing erythrocytes and grouped by genotype for the Trim33 mutation. (PDF) [file pgen.1005693.s002.pdf]

**A** Trim33 A301-060A  
(Bethyl Laboratories Inc.)  
Primary Antibody 1:10000

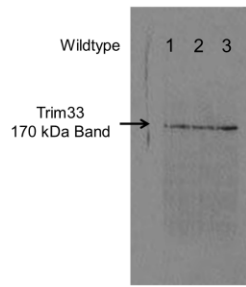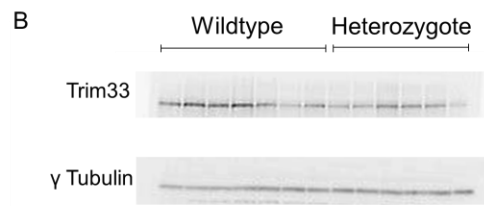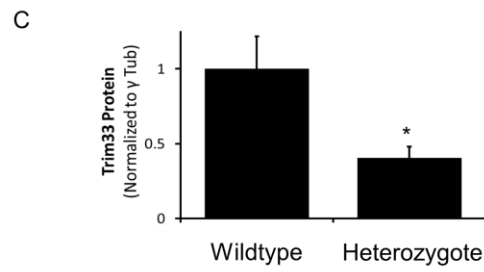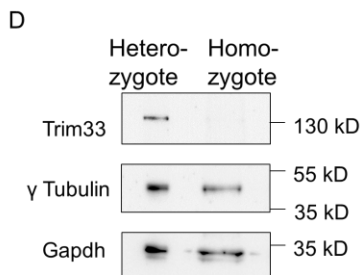

Supplement: S3 Fig — (A) The Trim33 antibody specificity at a 1:10000 dilution was tested using protein extract from three independent wildtype samples. The lysate was separated on a polyacrylamide gel. (B) Wildtype (n = 7) and heterozygote (n = 6) testis extracts incubated with the Trim33 antibody and an antibody against γ Tubulin. (C) Densitometry analysis of B shows Trim33 is significantly reduced in heterozygotes compared to wildtypes (p value < 0.05), error bars indicate SEM. (D) Heterozygote and homozygote protein extracts, from pooled E9.5 embryos (N = 4 per sample), incubated with the Trim33 antibody and antibodies directed against the housekeepers γ Tubulin and Gapdh. (PDF) [file pgen.1005693.s003.pdf]

## Wildtype testis

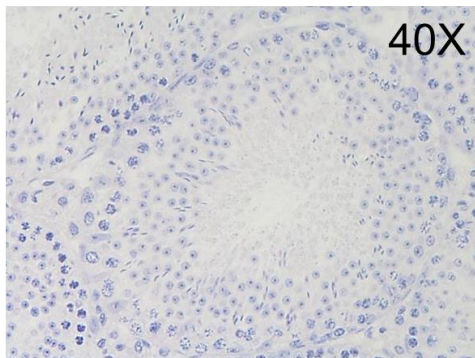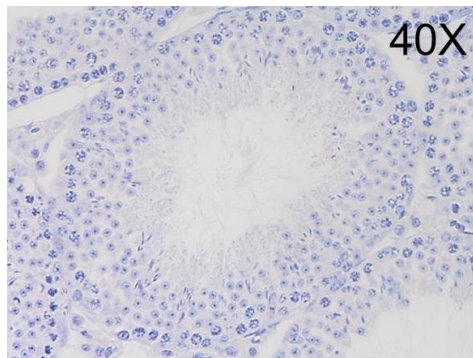

## *MommeD44* heterozygous testis

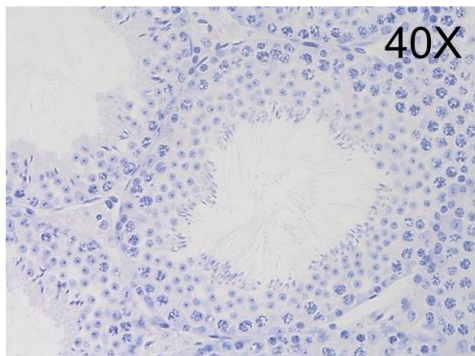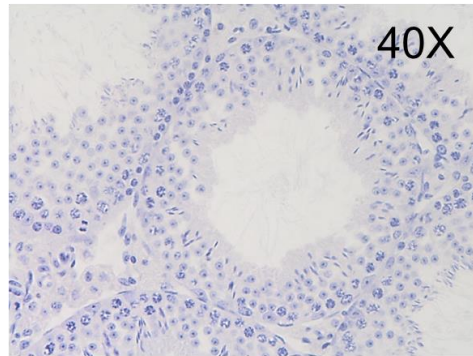

Supplement: S4 Fig — Mice heterozygous for MommeD44 show no obvious differences in histological sections after staining with haematoxylin, compared to MommeD44 wildtype mice. (PDF) [file pgen.1005693.s004.pdf]

A

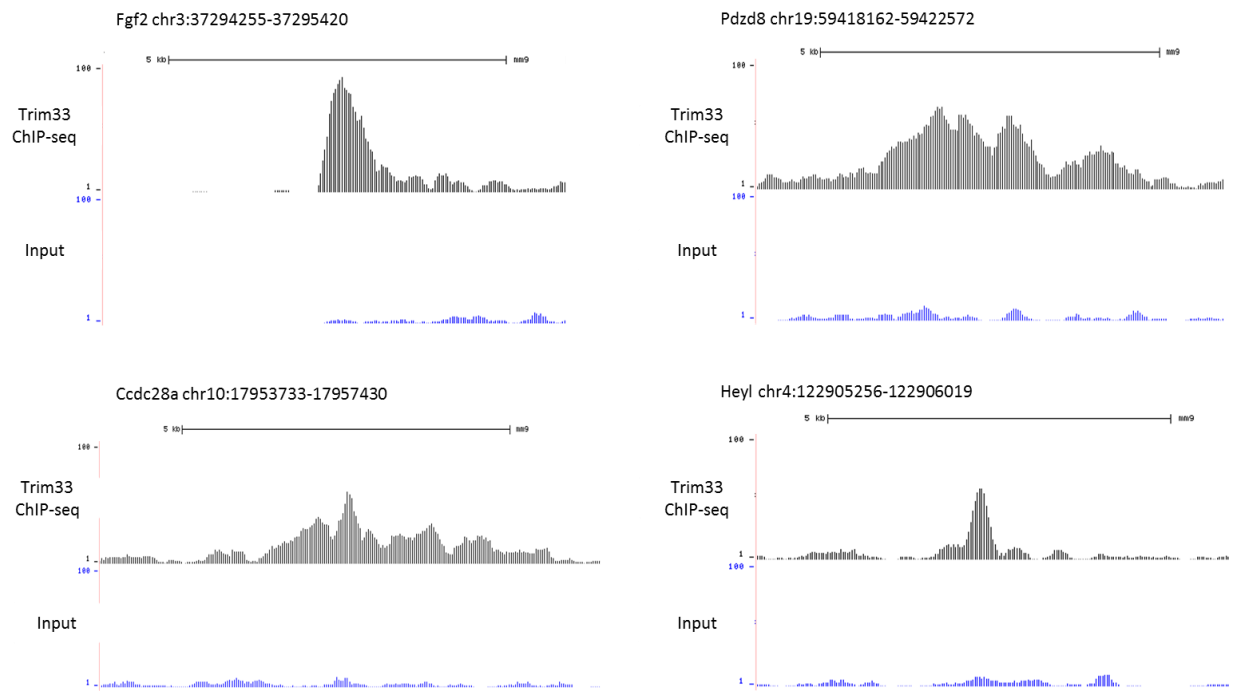

B

ChIP qPCR validation of ChIPseq data

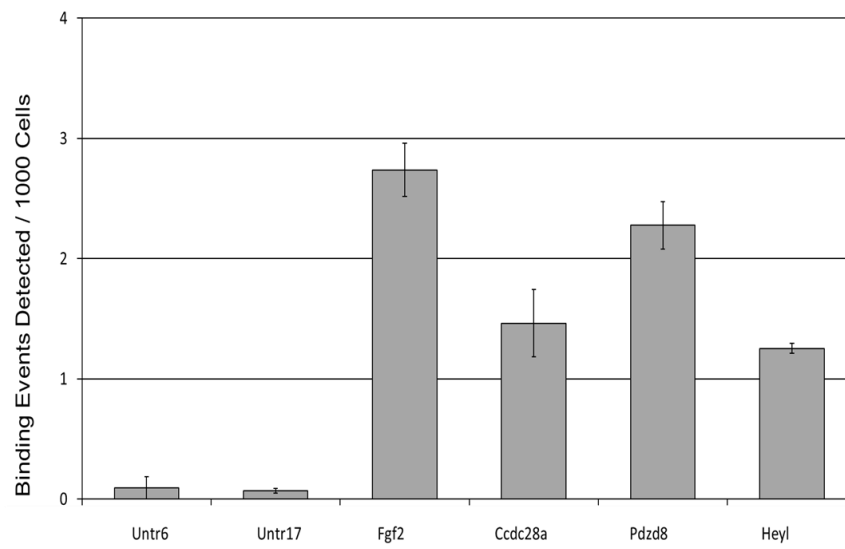

Supplement: S5 Fig — (A) Screenshots of Trim33 ChIP-seq (and Input) read density for four sites that were identified as Trim33 binding peaks. Shown are the 5kb loci centred on each of the peaks and labelled according to their distance from the nearest gene transcription start site: an intronic locus located within the Fgf2 gene, two gene promoter loci (Ccdc28a + Pdzd8) and an intergenic Trim33 binding site 5Kb upstream of the gene Heyl. The intronic locus within Fgf2 gene is located at an RLTR10B retrotransposon. (B) ChIP qPCR for six loci, two representing negative controls (Untr6 + Untr17) with primers located in regions not enriched for Trim33 and the four Trim33 enriched regions from A. Error bars indicate S.D. from 3 technical replicates. (PDF) [file pgen.1005693.s005.pdf]

A

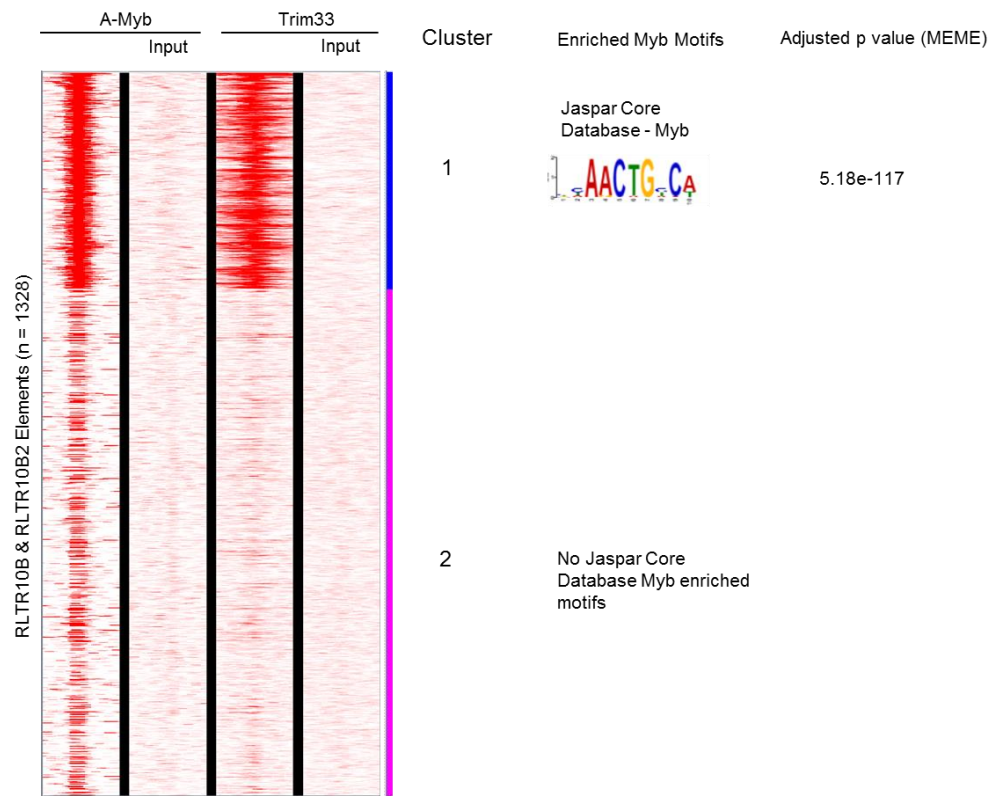

B

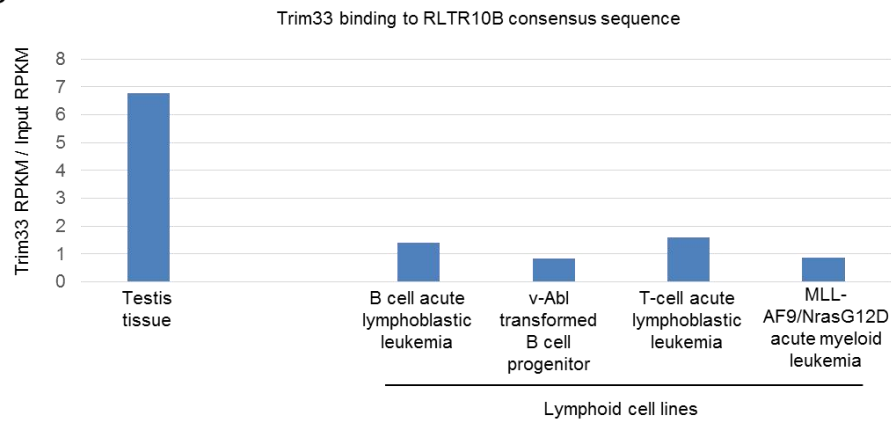

Supplement: S7 Fig — (A) Heat plot of ChIP-seq read density, clustered by similarity, for publically available ChIP-seq data for the A-Myb (plus input)–GEO accession GSE44690 and Trim33 and input in testis, at all RLTR10B and RLTR10B2 elements. Of the two clusters, only cluster 1 is enriched in the Jaspar Core Database Myb consensus binding sequence using the MEME program. (B) ChIP-seq data from another study [20], accession number GSE66233, was mapped to the RLTR10B RepBase consensus sequence. Enrichment values are from one ChIP-seq experiment per cell line as the fraction of the RPKM supporting binding compared to the input RPKM. Shown also is the ChIP-seq data from testis mapped to the RLTR10B element. (PDF) [file pgen.1005693.s007.pdf]

ERVK  
element E value

Jaspar Core Myb Consensus sequence  
Match with P value < 0.0001

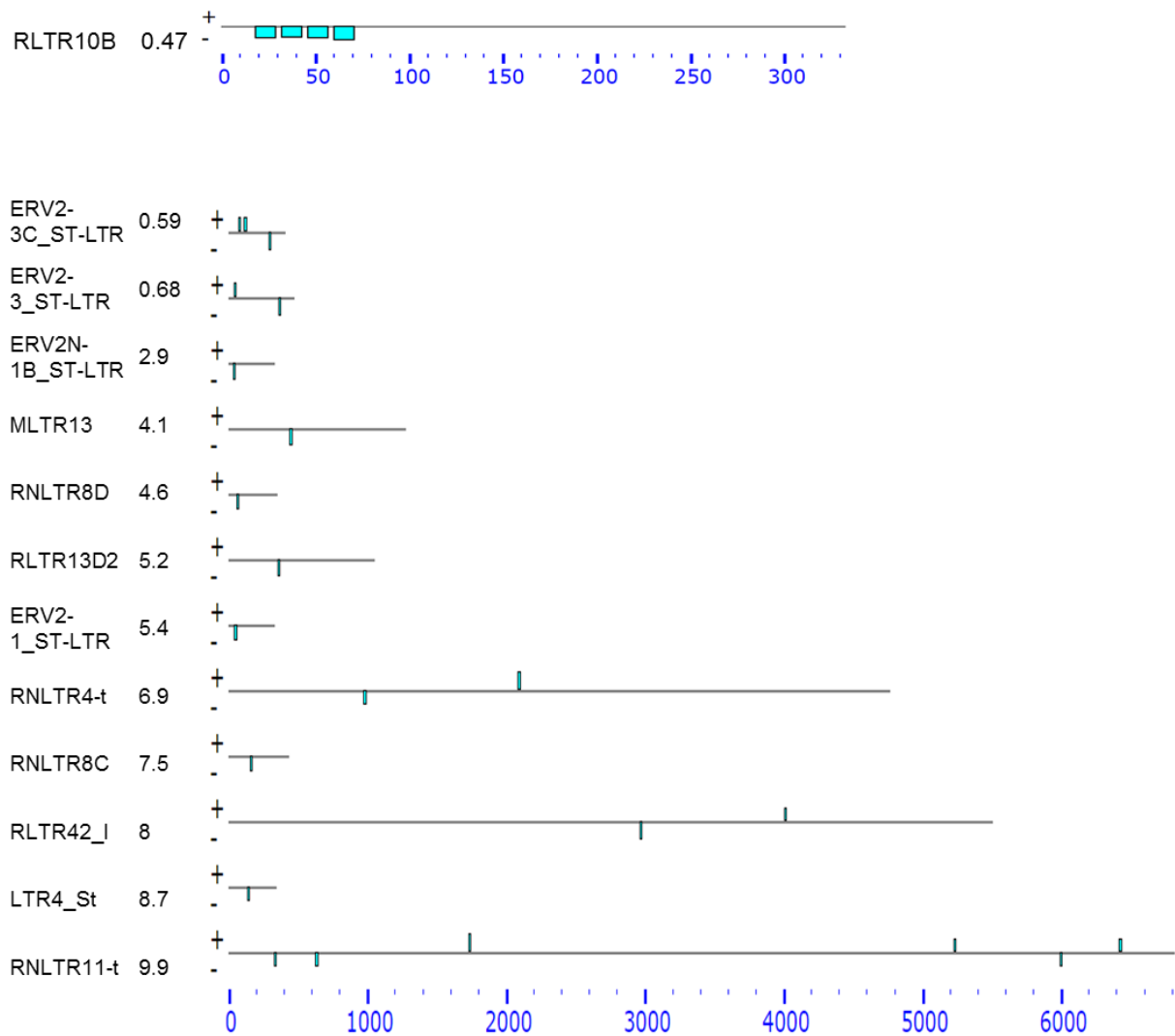

Supplement: S8 Fig — The MEME suite program MAST was used to search for Jaspar Core Database Myb consensus binding sites (shown in green) in the 447 ERV2 elements in the rodent RepBase database. Those elements with an E value of less than 10 are shown. The E value for each element is equal to the combined p-value of the sequence times the number of sequences searched. (PDF) [file pgen.1005693.s008.pdf]

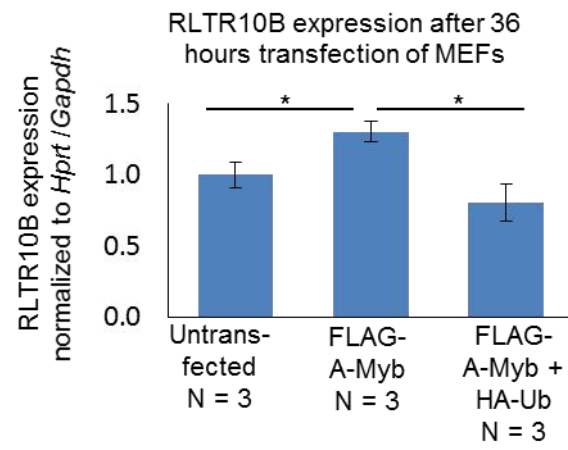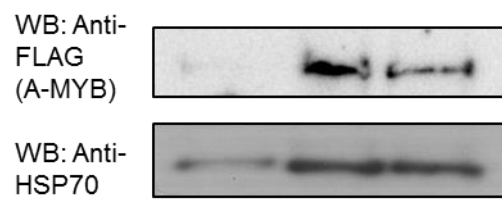

Supplement: S9 Fig — Immortalized MEF cells were transfected with FLAG-tagged A-MYB or FLAG-tagged A-MYB and HA-tagged ubiquitin for 36h. RLTR10B retrotransposon expression was measured and normalized to the expression of housekeeper genes Hprt and Gapdh. RLTR10B expression increased when A-MYB was overexpressed and the effect is reversed when ubiquitin was also overexpressed. Error bars indicate SEM, * p-value > 0.05. The expression levels of Flag-A-MYB in MEFs and HSP70 are shown (bottom panel). (PDF) [file pgen.1005693.s009.pdf]

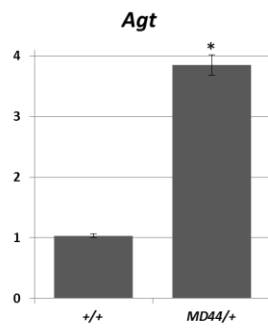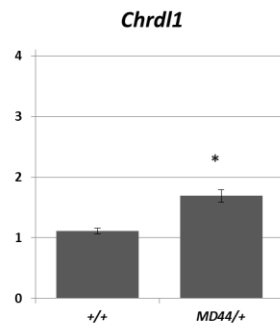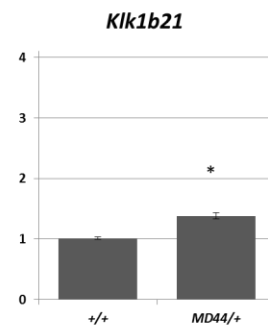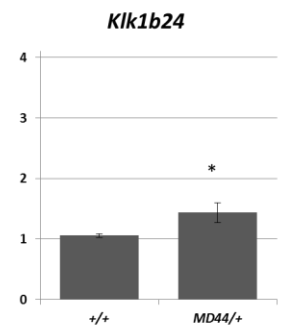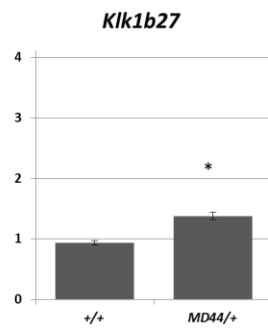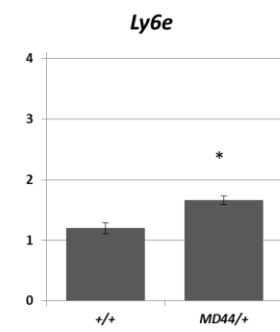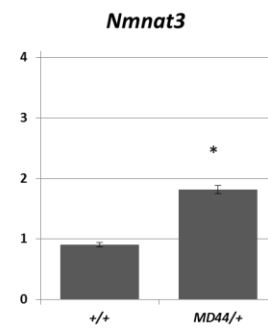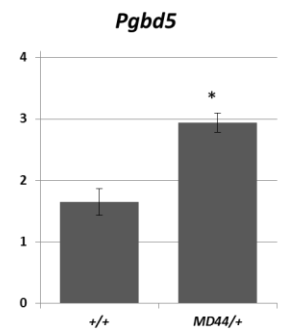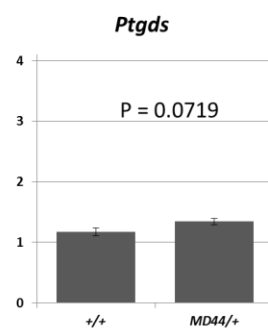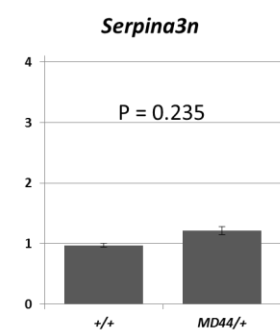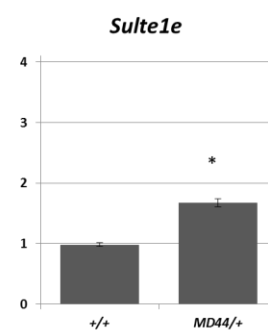

Supplement: S10 Fig — The expression for genes predicted to be significantly differentially regulated by RNA-seq (Fig 5) was tested by RTqPCR. Genes that displayed high expression in testis (greater than 100 read alignments) and fold change of at least 1.4X were chosen. Expression of the indicated genes was measured using primers to the transcript (across exon junctions) and normalized to two housekeeping genes, Hprt and Rps2, the number of samples for each group was at least 4, * p value ≤ 0.05, error bars = SEM. Ptgds and Serpina3n did not validate as significantly different between wildtypes and heterozygotes. (PDF) [file pgen.1005693.s010.pdf]

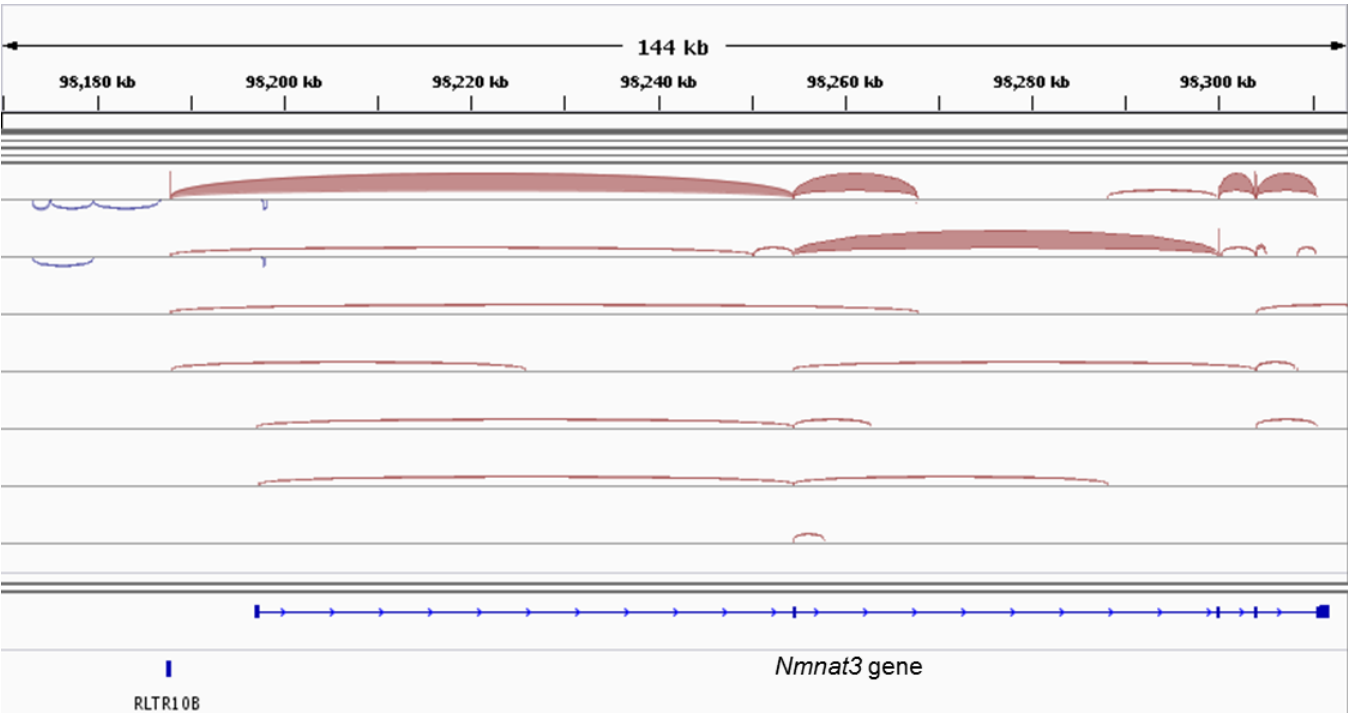

Supplement: S11 Fig — Splice junction tracks generated by the IGV genome browser program are shown. Each of the tracks represents a mRNA sequence built by the IGV browser program from the split reads mapped by the testis RNA-seq data. The thickness of the bands indicates the relative coverage of split reads supporting usage of a particular splice site. The majority of mRNA across the Nmnat3 locus begins at the RLTR10B element upstream. (PDF) [file pgen.1005693.s011.pdf]

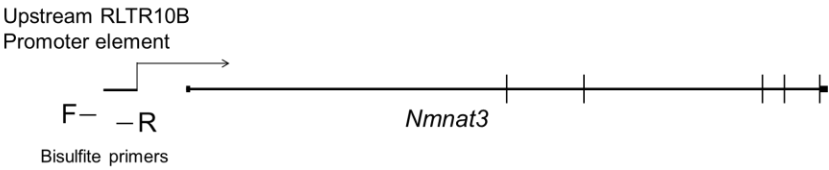

**Adult testis**

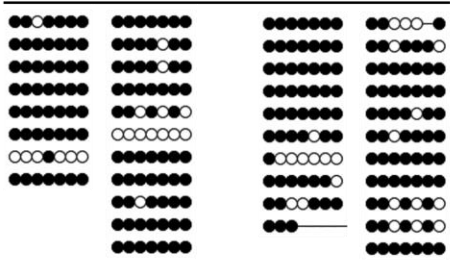

85% methylated  
(113/133)

84% methylated  
(119/142)

Supplement: S12 Fig — DNA methylation was measured inside the RLTR10B that functions as an upstream promoter of the Nmnat3 gene in testis. Bisulfite primer design inside the locus is indicated. The methylation state of individual animals (two per genotype) is shown as columns of cloned PCR products. Filled in circles represent methylated CpGs and white circles represent unmethylated CpGs. Each mouse is represented by eight or greater clones and averages for each sample and the two groups are indicated. The bisulfite conversion rate of the non CpG cytosines is indicated as 99%. (PDF) [file pgen.1005693.s012.pdf]

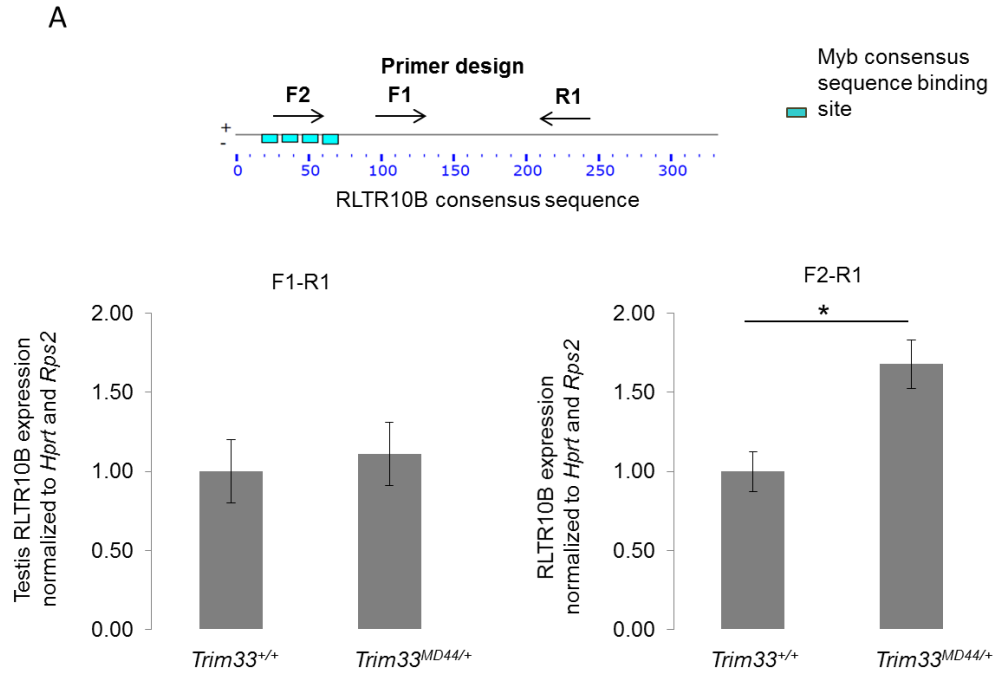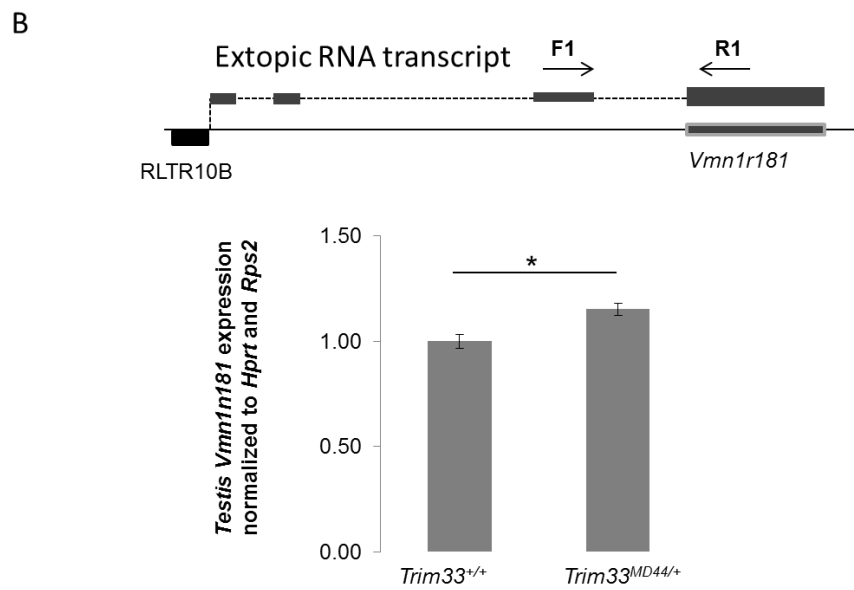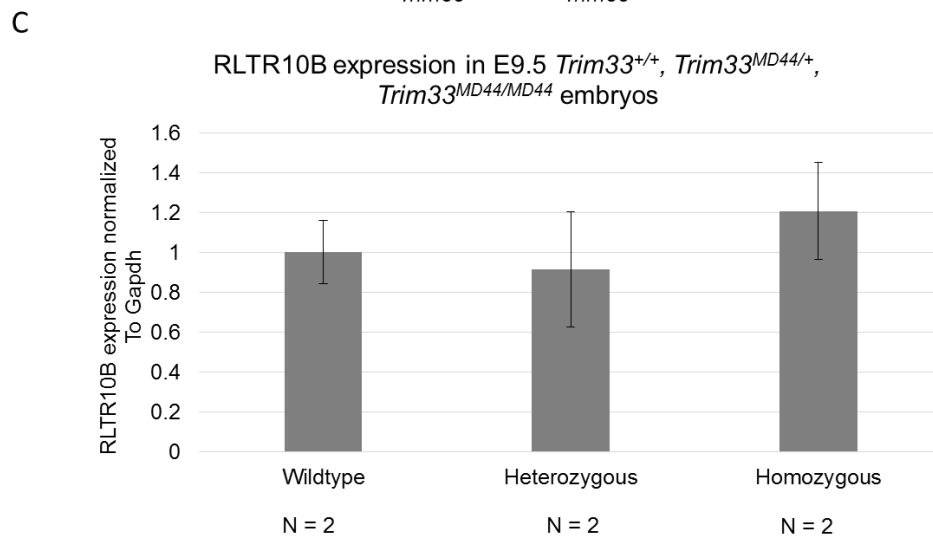

Supplement: S13 Fig — (A) Expression of RLTR10B elements in testis was measured using primer pairs that either included the Myb binding sites (F2 –R1) or not (F1 –R1). Increased expression of RLTR10B elements containing the Myb binding sites was seen in MommeD44 heterozygotes. Expression was normalized to two housekeeping genes, Hprt and Rps2, and then normalised to the average expression levels from wildtype mice. The number of samples for each group was at least 3, * p value ≤ 0.05, error bars = SEM. (B) Expression of an ectopic transcript, originating at an RLTR10B upstream of the Vmn1r181 gene, was measured in MommeD44 heterozygous and wildtype testis. Primer annealing locations are shown. Increased expression was detected in heterozygous tissue, normalized to the housekeeping genes Hprt and Rps2, * p value ≤ 0.05, error bars = SEM. (C) Expression of RLTR10B elements was measured in E9.5 embryos from the same litter; wildtype, heterozygous or homozygous for the MommeD44 mutation. No change in expression was detected across genotypes, normalized to the housekeeping gene Gapdh. (PDF) [file pgen.1005693.s013.pdf]

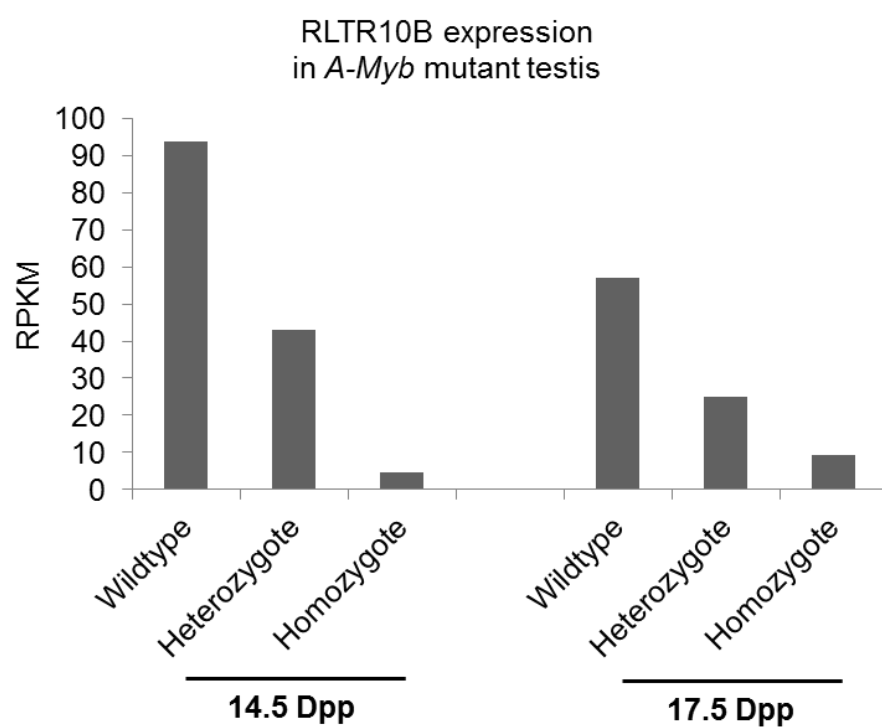

Supplement: S14 Fig — RNA-seq data from another study [18], accession number GSE44690, was mapped to the RLTR10B RepBase consensus sequence. RPKM values are from one biological replicate per genotype for each time point. (PDF) [file pgen.1005693.s014.pdf]
