# Supplementary material for: Trim33 Binds and Silences a Class of Young Endogenous Retroviruses in the Mouse Testis; a Novel Component of the Arms Race between Retrotransposons and the Host Genome
Source: PLoS Genet. 2015 Dec 1;11(12):e1005693. doi: 10.1371/journal.pgen.1005693 (PMC4666613; doi:10.1371/journal.pgen.1005693)
Supplement: S6 Fig — Several motifs were significantly enriched in highly significant (P value < 1.0e-20) Trim33 ChIP-seq peaks (N = 2338) that were significantly homologous to known DNA binding consensus sequences from the JASPAR CORE 2014 database (PDF) [file pgen.1005693.s006.pdf]

Significant motifs (E-value ≤ 0.05) identified in Trim33 ChIP seq peaks, using the program MEME

Enriched motifs similar to known transcription factors		E-value	Distribution
MYB (MA0100.2)	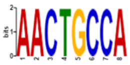	1.2e-22	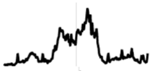
ZEB1 (MA0103.2)	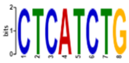	3.0e-16	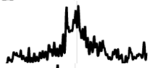
NRF1 (MA0506.1)	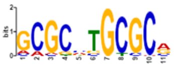	5.8e-5	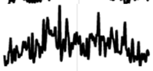
Zfx (MA0146.2)	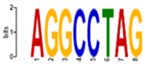	1.6e-4	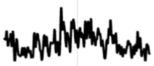
NFYB (MA0502.1)	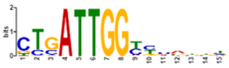	1.4e-3	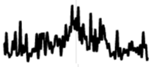
NFYA (MA0060.2)	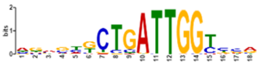	2.0e-2	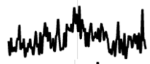
THAP1 (MA0597.1)	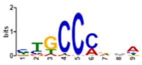	2.4e-2	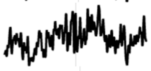
EGR1 (MA0162.2)	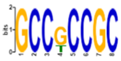	1.5e-2	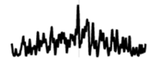
